# Intranasal sufentanil versus intravenous morphine for acute severe trauma pain: A double-blind randomized non-inferiority study

**DOI:** 10.1371/journal.pmed.1002849

**Published:** 2019-07-16

**Authors:** Marc Blancher, Maxime Maignan, Cyrielle Clapé, Jean-Louis Quesada, Roselyne Collomb-Muret, François Albasini, François-Xavier Ageron, Stephanie Fey, Audrey Wuyts, Jean-Jacques Banihachemi, Barthelemy Bertrand, Audrey Lehmann, Claire Bollart, Guillaume Debaty, Raphaël Briot, Damien Viglino

**Affiliations:** 1 Emergency Department and Mobile Intensive Care Unit, Grenoble Alpes University Hospital, Grenoble, France; 2 HP2 Laboratory, INSERM U1042, University Grenoble Alpes, Grenoble, France; 3 Clinical Pharmacology Unit, INSERM CIC1406, Grenoble Alpes University Hospital, Grenoble, France; 4 Emergency Department and Mobile Intensive Care Unit, Saint-Jean-de-Maurienne Hospital, Saint-Jean-de-Maurienne France; 5 Emergency Department, Centre Hospitalier Annecy Genevois, Annecy, France; 6 Emergency Department and Mobile Intensive Care Unit, Metropole Savoie Hospital, Chambery, France; 7 Emergency Department, Albertville–Moutiers Hospital, Moutiers, France; 8 Emergency Trauma Unit, Department of Orthopedic Surgery and Sport Traumatology, Hôpital Sud, Grenoble Alpes University Hospital, Grenoble, France; 9 Pharmacy Department, Grenoble Alpes University Hospital, Grenoble, France; 10 Clinical and Innovation Research Department, Grenoble Alpes University Hospital, Grenoble, France; 11 CNRS TIMC-IMAG Laboratory, UMR 5525, University Grenoble Alpes, Grenoble, France; UT-Southwestern Medical Center, UNITED STATES

## Abstract

**Background:**

Intravenous morphine (IVM) is the most common strong analgesic used in trauma, but is associated with a clear time limitation related to the need to obtain an access route. The intranasal (IN) route provides easy administration with a fast peak action time due to high vascularization and the absence of first-pass metabolism. We aimed to determine whether IN sufentanil (INS) for patients presenting to an emergency department with acute severe traumatic pain results in a reduction in pain intensity non-inferior to IVM.

**Methods and findings:**

In a prospective, randomized, multicenter non-inferiority trial conducted in the emergency departments of 6 hospitals across France, patients were randomized 1:1 to INS titration (0.3 μg/kg and additional doses of 0.15 μg/kg at 10 minutes and 20 minutes if numerical pain rating scale [NRS] > 3) and intravenous placebo, or to IVM (0.1 mg/kg and additional doses of 0.05 mg/kg at 10 minutes and 20 minutes if NRS > 3) and IN placebo. Patients, clinical staff, and research staff were blinded to the treatment allocation. The primary endpoint was the total decrease on NRS at 30 minutes after first administration. The prespecified non-inferiority margin was −1.3 on the NRS. The primary outcome was analyzed per protocol. Adverse events were prospectively recorded during 4 hours. Among the 194 patients enrolled in the emergency department cohort between November 4, 2013, and April 10, 2016, 157 were randomized, and the protocol was correctly administered in 136 (69 IVM group, 67 INS group, per protocol population, 76% men, median age 40 [IQR 29 to 54] years). The mean difference between NRS at first administration and NRS at 30 minutes was −4.1 (97.5% CI −4.6 to −3.6) in the IVM group and −5.2 (97.5% CI −5.7 to −4.6) in the INS group. Non-inferiority was demonstrated (*p <* 0.001 with 1-sided mean-equivalence *t* test), as the lower 97.5% confidence interval of 0.29 (97.5% CI 0.29 to 1.93) was above the prespecified margin of −1.3. INS was superior to IVM (intention to treat analysis: *p =* 0.034), but without a clinically significant difference in mean NRS between groups. Six severe adverse events were observed in the INS group and 2 in the IVM group (number needed to harm: 17), including an apparent imbalance for hypoxemia (3 in the INS group versus 1 in the IVM group) and for bradypnea (2 in the INS group versus 0 in the IVM group). The main limitation of the study was that the choice of concomitant analgesics, when they were used, was left to the discretion of the physician in charge, and co-analgesia was more often used in the IVM group. Moreover, the size of the study did not allow us to conclude with certainty about the safety of INS in emergency settings.

**Conclusions:**

We confirm the non-inferiority of INS compared to IVM for pain reduction at 30 minutes after administration in patients with severe traumatic pain presenting to an emergency department. The IN route, with no need to obtain a venous route, may allow early and effective analgesia in emergency settings and in difficult situations. Confirmation of the safety profile of INS will require further larger studies.

**Trial registration:**

ClinicalTrials.gov NCT02095366.

EudraCT 2013-001665-16.

## Abstract 2

In the emergency departments of six French hospitals, Marc Blancher and team compared intranasal sufentanil versus intravenous morphine for acute severe trauma pain in a randomized, double-blind, non-inferiority trial.

## Author summary

**Why was this study done?:**

- To relieve acute pain in an emergency room, the priorities are analgesia as early as possible, using an agent with rapid onset of action and powerful enough to achieve a level of pain as low as possible.
- Intravenous morphine is the most commonly used strong analgesic, but the need to obtain intravenous access is a clear limitation to its rapid use.
- The intranasal route using sufentanil spray could provide rapid easy administration with fast peak action time.

**What did the researchers do and find?:**

- We compared the effectiveness of intranasal sufentanil to that of intravenous morphine for patients presenting at an emergency room with acute severe traumatic pain.
- Patients were randomly assigned to receive intranasal sufentanil and intravenous placebo, or intravenous morphine and intranasal placebo.
- The 2 treatments gave similar levels of relief from pain according to self-assessments of pain by the patients.

**What do these findings mean?:**

- In the emergency room, intranasal sufentanil is feasible and not inferior to intravenous morphine in reducing severe pain due to traumatic injury at 30 minutes.
- Adverse events were frequent, as expected for opioids, but rarely serious. Larger studies are needed to confirm safety.
- The use of intranasal sufentanil might provide an easy and time-saving solution in the management of acute pain.

## Introduction

Pain is the most common symptom presented by patients in hospital emergency departments, particularly in the context of trauma. It is estimated that fewer than half of patients receive analgesics within 1 hour of arrival [1–3]. Despite the wide range of therapeutic methods available, their prompt administration is hindered by the availability of the necessary material conditions. The 3 priorities for emergency acute pain treatment are implementation as early as possible, rapid onset of action, and powerful enough to reach a level of pain as low as possible. Intravenous morphine (IVM) is the most common strong analgesic, but is associated with a clear time limitation related to the need to obtain an access route. Inhaled, intramuscular, and sublingual pathways have been tested for many years with gaseous molecules or adapted formulations [4–7]. The intranasal (IN) route provides easy administration with a fast peak action time due to high vascularization and the absence of first-pass metabolism [8–10]. Many molecules have been evaluated, but they need to be at high concentrations (small volume vaporized) and liposoluble to be effective. Despite studies on fentanyl showing encouraging results and adequate IN-to-systemic pharmacokinetics [10–12], its use is still scarce in emergency practice. Sufentanil has the advantage of being a stronger opioid, well known by emergency physicians and intensivists, and available in high concentration, with known rapid peak action time (about 5 minutes) via an intravenous (IV) route [13]. However, the short-term analgesic action of sufentanil administered by a nasal route on acute severe pain is still poorly evaluated [14–17]. Apart from non-randomized and open-label studies [14,15], 2 recent studies evaluated IN sufentanil (INS) in good methodological conditions. Lemoel et al. [16] compared INS to venous analgesia but without a standardized comparator: the venous analgesia did not systematically include an opiate. Sin et al. [17] performed a low-powered study (30 patients) and showed no difference in analgesia, but the study was not designed to prove non-inferiority. Our aim was therefore to assess the non-inferiority of the analgesic effect and safety of titrated INS compared to titrated IVM administered to patients with non-life-threatening trauma.

## Methods

### Study design and participants

The ALGOFINE trial was designed as a multicenter, double-blind, randomized, controlled non-inferiority trial. The full protocol for this trial is available as S1 Text. Adult patients (18 to 75 years old) presenting with traumatic pain self-evaluated as ≥6/10 on a numerical pain rating scale (NRS) were recruited at triage in 6 hospital emergency departments (Grenoble Alpes University Hospital [Grenoble], Metropole-Savoie Hospital [Chambery], Centre Hospitalier Annecy Genevois [Annecy], Albertville–Moutiers Hospital [Albertville], Saint-Jean-de-Maurienne Hospital [Saint-Jean-de-Maurienne], and Voiron Hospital [Voiron]). Patients were not enrolled if they presented with 1 of the following criteria at triage: pain of non-traumatic origin; chronic respiratory, renal, or hepatic insufficiency; any history of drug addiction; past medical or surgical sinus pathology; oxygen saturation < 90%; systolic blood pressure < 90 mm Hg; traumatic brain injury with a Glasgow Coma Scale score < 14; opioid allergy; facial trauma; patient unable to understand or self-assess using a NRS; opiate administration within 6 hours before admission; or weight > 100 kg. Information regarding procedures, possible adverse events or inefficacy, and data privacy was given to the participants before obtaining signed consent. In particular, participation in the study was proposed during the first few minutes of triage, given the urgency of their need for pain relief. They were informed that in view of their level of pain, IVM was normally indicated, and would be offered by the doctor in charge. An explanation regarding sufentanil, IN administration, and the principles of randomization and blinding were given while the nurse in charge obtained the venous route required even if the patient declined to participate.

A group of patients recruited in a pre-hospital setting by a mobile emergency unit was planned. They are not included in the present study population, and the results of this prespecified ancillary study are not described here (at the reviewers’ request).

The study was investigator initiated and conducted in accordance with the principles of the Declaration of Helsinki. The protocol (EudraCT 2013-001665-16; ClinicalTrials.gov NCT02095366) was approved by the regional ethics board on May 15, 2013 (Rhone-Alpes Auvergne Clinical Research Centers ethics committee CECIC IRB N° 2013-001665-16), the national commission for liberties and data protection (Commission Nationale de l'Informatique et des Libertés), and the national drug safety agency (Agence Nationale de Sécurité du Médicament et des Produits de Santé). All information regarding procedures, risks, and data privacy was made known to the participants before obtaining signed consent. This study is reported as per the Consolidated Standards of Reporting Trials (CONSORT) guideline (see S1 CONSORT Checklist).

### Randomization and masking

All clinical and research staff as well as patients were blinded to the treatment allocation except for the nurse in charge of the preparation of the treatments. Treatments were prepared from randomized packs in a dedicated room, and syringes labeled only “IV” and “IN” were given to the investigator. All study drugs were packaged in blinded trial packs by a clinical trial pharmacist who was blinded to interventions and outcomes. Randomization (1:1) in parallel groups was pre-performed in blocks of random sizes and stratified by center using dedicated software hosted in Grenoble Alpes University Hospital. The randomization list was handed over to the central pharmacy in charge of preparing the packs.

### Procedures

At inclusion, and at 10 minutes and 20 minutes after inclusion if NRS remained >3, each patient received both a single dose of active agent and a single dose of placebo via the IV and IN routes. In the INS group, an initial dose of 0.30 μg/kg sufentanil (0.15 μg/kg in each nostril) was administered. Additional doses (0.15 μg/kg) were administered at 10 and 20 minutes in 1 of the nostrils if NRS remained >3/10. A stock solution at 50 μg/ml of sufentanil was sampled at each administration according to the patient’s weight with a conventional 1-ml syringe and atomized with a nasal mucosal atomization device (MAD, Wolfe Tory Medical, Salt Lake City, UT, US). The MAD is a simple device with a Luer lock tip that is readily connected to the syringe and provides complete vaporization of the contents. The MAD allows blinded procedures and precise dosage according to the patient’s weight since a standard syringe is used rather than a commercial ready-to-use system.

In the IVM group, an initial dose of 0.1 mg/kg was administered, and additional doses of 0.05 mg/kg at 10 and 20 minutes if the NRS remained >3/10. In both groups, placebo was 0.9% sodium chloride, administered either intravenously or by nasal atomization in the same volume that would have been given for the active treatment. During the first 30 minutes, the physician in charge was authorized to use co-analgesics, except for sedatives and strong opiates. After an outcome evaluation at 30 minutes, the physician in charge was informed of the nature and amount of drug administered in order to increase medication if necessary.

### Data collection and outcome measures

The primary endpoint was analgesia efficacy defined as a decrease in pain on the NRS between the first administration and 30 minutes later. Secondary endpoints included analgesia efficacy at 10 and 20 minutes defined by a decrease in NRS, and the incidence of any serious and non-serious adverse events up to 4 hours after the first administration. Vital parameters were collected every 10 minutes over 1 hour, including Ramsay Sedation Scale and Glasgow Coma Scale scores. Side effects and symptoms were recorded throughout the procedure, including those collected by posing specific questions to the patient every 10 minutes. The patient’s satisfaction regarding the procedure was assessed at 30 minutes.

Study data were collected and managed using the REDCap (Research Electronic Data Capture) electronic data capture tool hosted at Grenoble Alpes University Hospital [18]. REDCap (Research Electronic Data Capture) is a web-based application to support data capture, providing validated data entry, audit trails for tracking data processing and export, automated export procedures to download data to commonly used statistical packages, and procedures for importing data [18].

### Statistical analysis

#### Sample size, power, and statistical methods

As a non-inferiority study, the threshold was based on a clinically meaningful difference in efficacy (NRS_T30_ − NRS_T0_) between the 2 treatments of −1.3 [19,20]. From previous studies it was estimated that the standard deviation of the reduction in pain would be 2.8 [21]. With a sample size of 198 patients (99 in each group), a 2-group design would provide at least 80% power to reject the null hypothesis (corresponding to a loss of efficacy greater than or equal to 1.3 [19]), in favor of the alternative hypothesis, corresponding to a gain in efficacy or a loss of efficacy of less than 1.3, assuming that the expected mean difference was 0, the common standard deviation 2.8 [21], and the level of significance 2.5%. The target sample size was therefore set at 218 participants (109 in each arm) to account for potential protocol deviations or premature interruptions. The sample size calculation was performed using NQuery Advisor 7.0 software (Statistical Solutions, Boston MA, US).

#### Data analysis

Non-inferiority was determined on the basis of a 1-sided mean-equivalence *t* test (two 1-sided *t* tests approach, TOSTT procedure) [22] on the per protocol population (primary endpoint: NRS efficacy [NRS_T30_ − NRS_T0_]) and confirmed, for sensitivity reasons, on the modified intention to treat population, according to recommendations. Implementation of the modified intention to treat analysis required the replacement of missing data; this was performed using the multiple imputation method. Ten imputations of the missing data were used in the multiple imputation process. Following the reviewers’ recommendations, we performed a linear regression adjusted for baseline pain score and site.

We evaluated the superiority of the INS treatment over IVM at 30 minutes in the modified intention to treat population using a 2-sample Student *t* test. The superiority analysis was planned after the beginning of patient enrollment (and before any unblinding), if non-inferiority was met. We also compared the efficacy of the INS treatment to IVM treatment at the different time points (NRS_T10_ − NRS_T0_ and NRS_T20_ − NRS_T0_) in the intention to treat population using a 2-sample Student *t* test. We evaluated the efficacy of INS treatment and IVM at different times within each group (NRS_T0_ versus NRS_T10_ and NRS_T0_ versus NRS_T20)_ in the per protocol population and in the modified intention to treat population using a paired Student *t* test (or a Wilcoxon test if necessary). We compared the incidence of serious and non-serious adverse events between the groups using a chi-squared test, or a Fisher exact test if necessary (expected frequency less than 5). We summarized patient satisfaction (100-point scale) as median and interquartile range (IQR), compared using a Mann–Whitney test (the test used was not prospectively planned in the analysis plan). Statistical analyses were performed using Stata 13.1 software (StataCorp, College Station, TX, US).

## Results

### Patient characteristics

**Fig 1:**
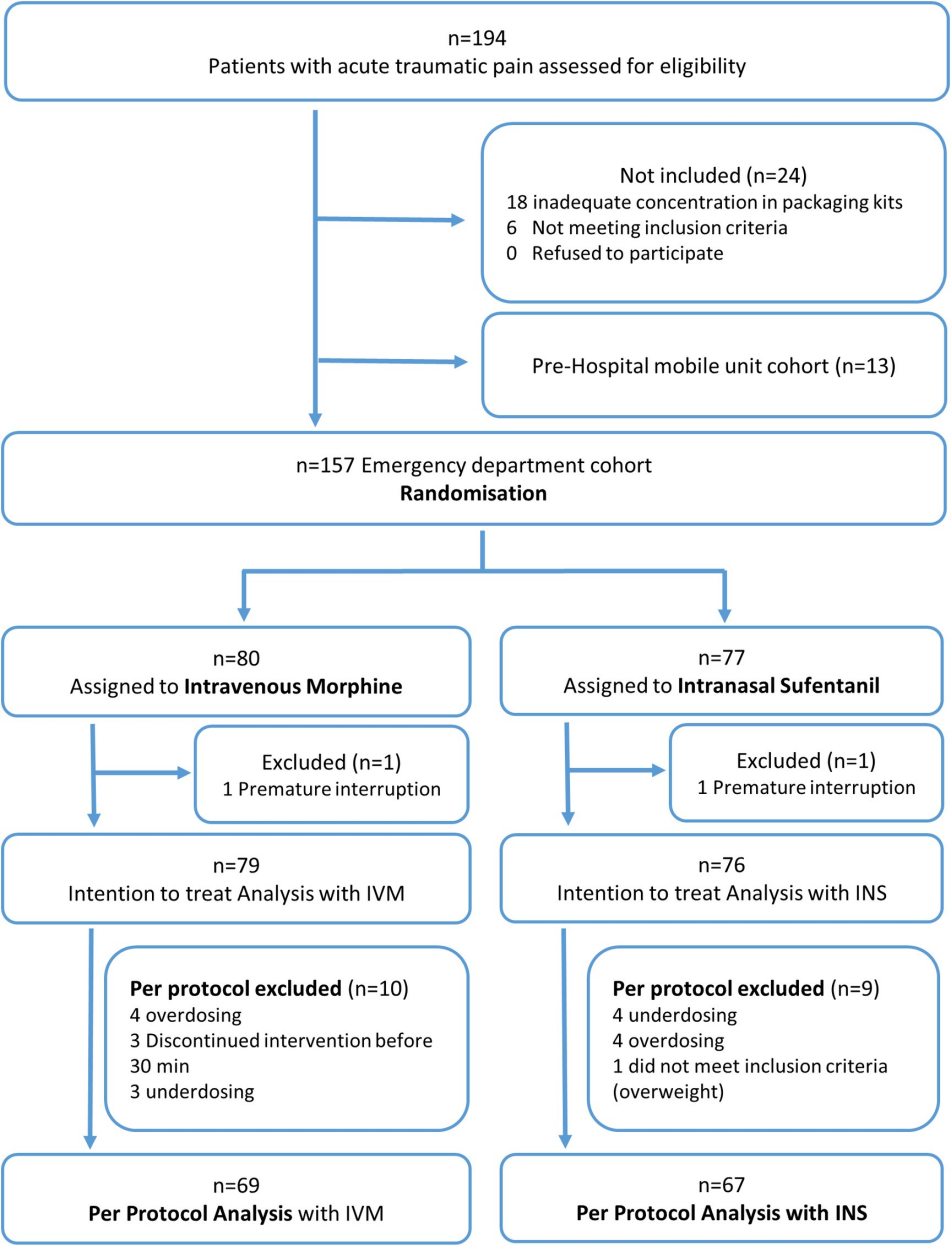
Enrollment, randomization, and follow-up of study participants. INS, intranasal sufentanil; IVM, intravenous morphine.

Between November 4, 2013 and April 10, 2015 we assessed a total of 194 patients presenting with acute traumatic pain for eligibility; 157 were included in the emergency department cohort and randomized, and 155 initiated the treatment. Nineteen patients were excluded from the per protocol analyses, mainly for underdosing (*n =* 7) or overdosing (*n =* 8) (see Fig 1 for detailed patient flow chart), yielding *n =* 69 for the IVM group and *n =* 67 for the INS group. Although recruitment was slower than expected over the planned duration of the study, the power remained above 80%, with an effective standard deviation in reduction in pain of 2.1 in post hoc analysis. The 2 treatment groups were well balanced with respect to baseline characteristics, with the exceptions of the male/female ratio and the number of patients who received other concomitant analgesia (IVM 33% versus INS 22%; Table 1). The mean NRS at baseline (T0) was 7.6 (95% CI 7.3–7.9) in the IVM group and 7.9 (95% CI 7.6–8.2) in the INS group. The median total dose of IVM administered was 12.5 mg (IQR 10.0 to 15.1), and the median total dose of INS was 36.0 μg (IQR 30.0 to 42.7). Ten minutes after the first administration, 59 (85.5%) patients in the IVM group received a second dose and 60 (89.6%) patients in the INS group received a second dose. Twenty minutes after the first administration, 42 (60.9%) patients in the IVM group received a third dose and 38 (56.7%) patients in the INS group received a third dose.

**Table 1:** Baseline characteristics of participants (per protocol).

Characteristic	IVM (*n =* 69)	INS (*n =* 67)
**Patient characteristic**		
Median [IQR] age (years)	41 [28 to 54]	38 [30 to 55]
Men	40 (58)	31 (46)
Median [IQR] weight (kg)	74 [61 to 83]	70 [60 to 80]
Trauma area*		
Head	2 (2.9)	1 (1.5)
Shoulder	7 (10.1)	12 (17.9)
Arm/elbow	12 (17.4)	9 (13.4)
Wrist or hand	17 (24.6)	8 (11.9)
Thorax wall	3 (4.4)	5 (7.5)
Rachis	12 (17.4)	8 (11.9)
Pelvis/hip	10 (14.5)	5 (7.5)
Leg/knee	8 (11.6)	13 (19.4)
Ankle or foot	11 (15.9)	13 (19.4)
**Vital parameters at inclusion**		
Median [IQR] HR (per minute)	76 [68 to 91]	74 [67 to 84]
Median [IQR] RR (per minute)	16 [15 to 19]	18 [15 to 20]
Median [IQR] SpO_2_ (%)	99 [97 to 100]	99 [97 to 100]
Median [IQR] MAP (mm Hg)	98 [91 to 104]	94 [85 to 103]
Median [IQR] NRS (/10)	8 [7 to 8]	8 [7 to 9]
Co-analgesia*	**23 (33)**	**15 (22)**
Paracetamol	22 (32)	15 (22)
Codeine	2 (3)	2 (3)
Ketoprofen	4 (6)	1 (1)
**Inclusion site**		
Grenoble (north site)	41 (59.4)	42 (62.7)
Grenoble (south site)	2 (2.9)	2 (3.0)
Saint-Jean-de-Maurienne	10 (14.5)	8 (11.9)
Annecy	9 (13.0)	8 (11.9)
Chambery	4 (5.8)	3 (4.5)
Albertville	3 (4.3)	3 (4.5)
Voiron	0 (0)	1 (1.5)

Values are numbers (percentages) unless stated otherwise.

*A patient could have multiple traumatized areas or concomitant analgesics.

HR, heart rate; INS, intranasal sufentanil; IQR, interquartile range; IVM, intravenous morphine; MAP, mean arterial pressure; NRS, numerical pain rating scale; RR, respiratory rate.

### Primary endpoint (non-inferiority)

The mean difference between NRS at first administration and NRS at 30 minutes was −4.1 (97.5% CI −4.6 to −3.6) in the IVM group and −5.2 (97.5% CI −5.7 to −4.6) in the INS group (per protocol analysis). Non-inferiority was met (*p <* 0.001), as the lower 97.5% confidence interval of 0.29 was greater than the prespecified non-inferiority margin of −1.3 (mean difference in NRS variation between groups 1.11, 97.5% CI 0.29 to 1.93).

In the intention to treat analysis, the mean NRS difference between first administration and at 30 minutes was −4.4 (97.5% CI −4.9 to −3.8) in the IVM group and −5.1 (97.5% CI −5.7 to −4.5) in the INS group. Non-inferiority was met (*p <* 0.001), as the lower 97.5% confidence interval of −0.05 was greater than the prespecified non-inferiority margin of −1.3 (mean difference of NRS variation between groups 0.74, 97.5% CI −0.05 to 1.54).

After adjusting for center (hospital) and the baseline level of pain, the difference in NRS reduction between groups was 0.87 (97.5% CI 0.19 to 1.54, *p =* 0.012).

### Secondary endpoints

#### Superiority

**Fig 2:**
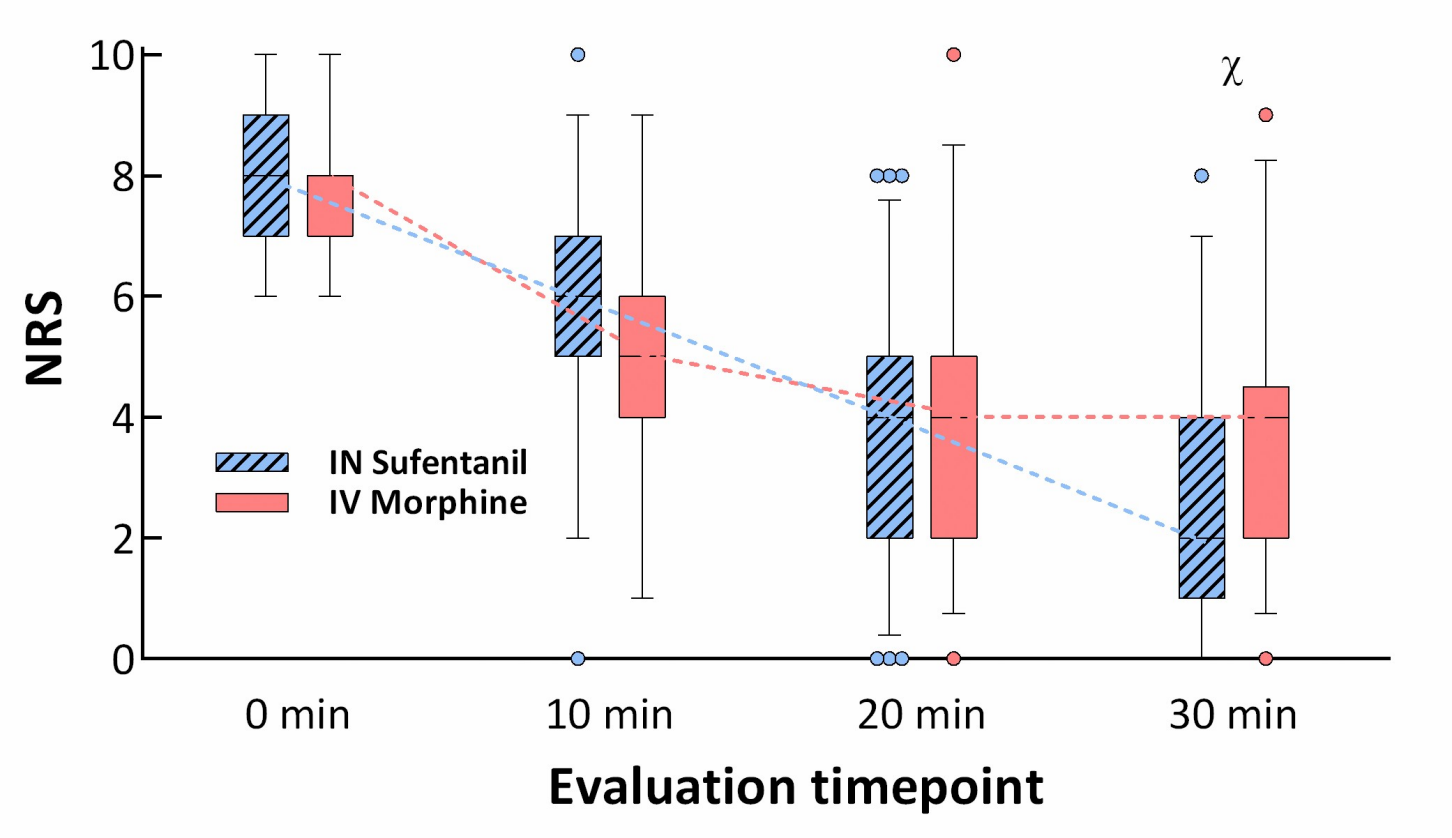
NRS at the different time points by group. The box extends from the 25th to the 75th percentile, and the whiskers are drawn down to the 5th percentile and up to the 95th. Points below and above the whiskers are drawn as individual points. No significant differences were observed in NRS values between groups at the 10-minute and 20-minute time points. IN sufentanil was non-inferior and superior to IV morphine in NRS reduction from drug administration to 30 minutes (NRS_T30_ − NRS_T0_). IN, intranasal; IV, intravenous; NRS, numerical pain rating scale.

**Fig 3:**
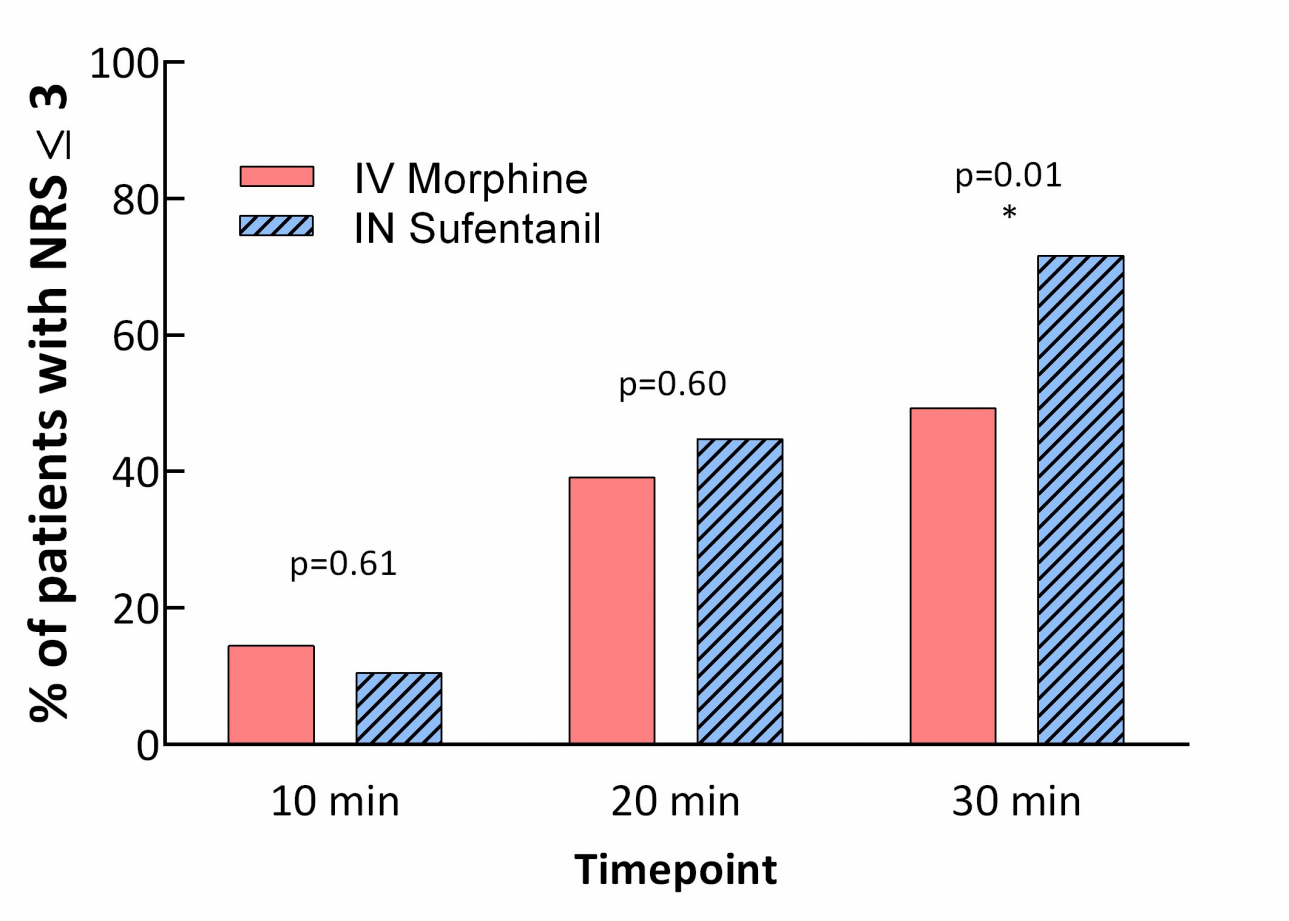
Patients with NRS ≤ 3 at the different time points by group. *Declared NRS was 3 or less at 30 minutes for 49.3% of patients in the IV morphine group and 71.6% in the IN sufentanil group (*p =* 0.01). IN, intranasal; IV, intravenous; NRS, numerical pain rating scale.

In superiority analysis, INS was superior to IVM for NRS pain reduction at 30 minutes in the intention to treat population (*p =* 0.034 for superiority), with a clinically nonsignificant difference in mean NRS between groups of 0.7. A significant decrease in NRS was observed after 10 minutes within both groups (mean difference −2.7, 95% CI −3.1 to −2.2, *p <* 0.001, for the IVM group and −2.1, 95% CI −2.5 to −1.7, *p <* 0.001, for the INS group, intention to treat), as well as after 20 minutes (mean difference −3.8, 95% CI −4.2 to −3.3, *p <* 0.001, for the IVM group and −4.0, 95% CI −4.5 to −3.5, *p <* 0.001, for the INS group, intention to treat). In a between-group comparison, no differences were found in NRS reduction at 10 and 20 minutes. Variations in NRS with time in each group are presented in Fig 2. In a subgroup analysis, self-assessed pain (NRS) was 3 or lower at 30 minutes for 34 (49.3%) patients in the IVM group and for 48 (71.6%) in the INS group (absolute difference = 22.3%, relative difference = 45.2%, *p =* 0.01; Fig 3). Sixteen patients in each group (23.2% in the IVM group and 23.9% in the INS group) received rescue pain medication by the physician in charge after unblinding.

#### Adverse events and patient satisfaction

There were no statistically significant differences between groups in recorded mild or serious adverse events in either the per protocol (Table 2) or the intention to treat populations. The most frequent adverse events observed were dizziness, hot flushes, and nausea or vomiting (Table 2). Hypoxemia (SpO_2_ < 90%) occurred in 3 (4.5%) patients in the INS group and in 1 (1.5%) patient in the IVM group. Bradypnea (respiration rate < 10/minute) was observed in 2 (3%) patients, both in the INS group. Finally, hypotension was reported in 1 (1.5%) patient in each group. None of the patients required naloxone, ventilation support, or vasopressors. Two patients required oxygen administration (3 l/min) during 10 minutes. The number of patients needed to harm was 17 (95% CI 7–56) for serious adverse events, including 33 (95% CI 12–38) for hypoxemia and 34 (95% CI 13–53) for bradypnea.

**Table 2:** Adverse events observed.

Event or symptom declared	IVM (*n =* 69)	INS (*n =* 67)	*p-*Value*
Severe adverse events	2 (2.9)	6 (9.0)	0.16
Hypoxemia (SpO_2_ < 90%)	1 (1.5)	3 (4.5)	0.36
Hypotension (SBP < 90 mm Hg)	1 (1.5)	1 (1.5)	1.00
Bradypnea (RR < 10/minute)	0 (0)	2 (3.0)	0.24
Anaphylactic shock	0 (0)	0 (0)	—
Alteration of consciousness (Ramsay > 2)	0 (0)	0 (0)	—
Bradycardia (bpm < 45/minute)	0 (0)	0 (0)	—
Naloxone use	0 (0)	0 (0)	—
Mild adverse events	42 (60.9)	31 (46.3)	0.09
Dizziness	25 (36.2)	19 (28.4)	0.33
Hot flushes	20 (29.0)	12 (17.9)	0.13
Nausea or vomiting	13 (18.8)	8 (11.9)	0.27
Bad taste/smell	3 (4.4)	2 (3.0)	1.00
Mild allergic reaction	1 (1.5)	1 (1.5)	1.00
Epistaxis/rhinorrhea	1 (1.5)	0 (0)	1.00
Hallucinations	0 (0)	0 (0)	—

Values are numbers (percentages).

*Fisher exact test or chi-squared test.

bpm, beats per minute; INS, intranasal sufentanil; IVM, intravenous morphine; RR, respiratory rate; SBP, systolic blood pressure.

The median patient satisfaction assessed on a 100-point scale at the end of the procedure was 80 (IQR 60 to 92.5) in the IVM group and 80 (IQR 70 to 100) in the INS group (*p =* 0.34).

## Discussion

In this randomized, double-blind trial comparing IV and IN opioid analgesia in emergency departments, INS was feasible and non-inferior to IVM in reducing severe pain from traumatic injury, at 30 minutes. Furthermore, the results suggest that the INS regimen (0.30 μg/kg and then 0.15 μg/kg every 10 minutes) is superior to IVM (0.1 mg/kg and then 0.05 mg/kg every 10 minutes) at 30 minutes, with a median decrease in NRS > 5, without, however, reaching a between-group difference in NRS reduction large enough to be considered clinically significant (i.e., a difference in NRS ≥ 1.3) [19,20]. The analgesic effectiveness of INS was observed after the first 10 minutes, with a mean within-group reduction in NRS of 2.1. Adverse events were frequent, as expected for opioids, but rarely serious, with no significant difference between the 2 groups, despite an imbalance observed to the detriment of INS.

So far, the use of INS has been mainly studied in palliative or pediatric patients, mostly in a perioperative context and in combination with other methods of sedation [23–29]. Only a few studies [14–17] have tested INS for acute pain management in the context of emergency practice. Two studies were conducted as non-randomized open-label trials [14–15], making the interpretation of efficacy and side effects difficult. In these 2 studies, 15 and 40 patients suffering from distal extremity injury received a single dose of 0.5 μg/kg of INS. The average pain score at 30 minutes was lowered by 4.3 points [14] and 4.7 [15], respectively. Some adverse effects were reported, such as dysphoria (46.6%), nausea (13.3%), and dizziness (7.5%), but no apnea was observed. More recently 2 monocentric randomized controlled trials versus placebo have been published [16–17]. Lemoel et al. [16] administered a single dose of 0.4 μg/kg of INS (or placebo) to 144 patients admitted to the emergency department for a recent (<6 h) isolated limb injury. Usual IV pain treatment was given to every patient, with multimodal analgesics including IV opioids if needed. The percentage of patients with satisfactory pain relief (NRS ≤ 3) was better in the sufentanil group (72.2%) than in the control group (51.4%). However, a higher rate of side effects was observed in the sufentanil group (12.5% more bradypnea and 24% more nausea) than in controls. This is probably due to the cumulative administration of opioids in the active group, who could receive both INS and IVM. Sin et al. [17] compared the administration of a single dose of 0.7 μg/kg of INS versus 0.1 mg/kg of IVM in 60 adult patients who presented to the emergency department with acute pain. They did not find any difference in NRS between the 2 groups 10 minutes after drug administration. Despite the high dose of sufentanil they used, they reported that only 7.5% of cases had dizziness, and none had dysphoria or apnea. All of these published studies considered the effects of a single administration of INS. Our protocol aimed to respect the principle of opioid titration, with repeated administrations of divided doses. Our patients received an average total dose of 36 μg of sufentanil in comparison with the average dose of 37.7 μg administered by Sin et al. [17]. Surprisingly, despite our titration strategy, we reported more frequent side effects than Sin et al. This might be due to the small number of patients they studied and also to the attention we paid to ensuring we collected every side effect in our non-inferiority design protocol.

Indeed, no studies to our knowledge to date have been methodologically designed to study the non-inferiority of INS versus IVM in terms of analgesic efficacy and side effects for trauma in adults, which is the most frequent situation encountered by emergency physicians. We designed our study to assess a strong, fast-acting opioid, hoping to observe a rapid and clear action that we could compare with morphine from the first 5 minutes onwards. In a titration protocol comparable to that commonly used for IVM, both non-inferiority and superiority were observed for INS at 30 minutes. The first 30 minutes is the time usually needed to obtain an IV cannula; this duration is shorter than the time of action of most other analgesics, other than inhaled therapies such as nitrous oxide or methoxyflurane [4,30]. Moreover, the effect of INS appears convincing as early as 5 minutes after administration, with a reduction in pain comparable to that obtained with IVM. The duration of action of sufentanil is short and depends on the route of administration used. Intravenously it has a half-life of about 15 minutes; this half-life is longer by a sublingual route due to further absorption [31,32]. When administered intranasally, the effect is probably intermediate [10,11], but remains short and results in an expected analgesia of about 45 minutes’ duration. Therefore, INS could be useful for the initiation of analgesia aimed at easing the pain as quickly as possible. In the context of the growing “opioid epidemic,” this mode of use could have the advantage of reducing the prescription of subsequent oral opioids responsible for addiction [33]. Overall, our results argue in favor of very early relief of pain during emergency triage that avoids conventional routes. The use of INS might become a pragmatic option in all situations where obtaining a venous access is a challenge or delays pain management. Thus, INS could be an alternative to IV analgesia in the pre-hospital setting, when IV access is not feasible or very difficult to obtain.

This randomized and double-blinded study should minimize most of the common biases. Nevertheless, our study has a number of limitations. Despite the random assignment, there was imbalance between the study arms in the male/female ratio and in the concomitant analgesics used. Allowing co-analgesia only after 30 minutes could have avoided this bias. On the other hand, opioid drugs are generally used as co-analgesics, in combination with acetaminophen in particular. From this point of view, the chosen methodology was closer to what happens in real clinical practice. As co-analgesia was more often used in the IVM group, this imbalance might have reduced the observed efficacy of INS compared to IVM but does not call into question the interpretation of non-inferiority. Although recruitment was lower than expected over the planned duration of the study, the power remained above 80%, with an effective standard deviation in reduction in pain lower than planned. The relatively low rate of participant inclusion was due to the difficulty of implementing a randomized trial during triage of patients experiencing intense pain. Finally, the study was not powered to answer the question of safety. Although the number of severe events observed was not statistically different between the 2 treatment groups, we observed an imbalance between the groups. The confirmation of safety would require a much larger trial. Future studies will also need to ensure that the use of INS is effective and safe in other emergency settings, such as pre-hospital or in difficult situations, such as in mountain rescue.

In conclusion, our results suggest that IN titration of sufentanil starting at an initial dose of 0.3 μg/kg is non-inferior to IVM for trauma-related pain reduction over the first 30 minutes. Moreover, the findings from our trial suggest that INS may be superior to IVM within this timeframe, although our results did not show a clinically significant difference. The IN route, with no need to obtain a venous route, may allow faster initiation of effective analgesia. Further larger studies are needed to determine the safety profile of INS.

## Supporting information

S1 CONSORT checklist This file contains the CONSORT checklist indicating where in the paper each CONSORT element is located. (PDF)

S1 Text Study protocol. This file contains the complete prospective study protocol, translated from the French version approved by the regional ethics board on May 15, 2013 (Rhone-Alpes Auvergne Clinical Research Center ethics board CECIC IRB N° 2013-001665-16).
(PDF)

S2 Text Final prospective analysis plan. (PDF)

S1 Dataset Complete dataset to replicate the analyses. The dataset includes participant characteristics and vital parameters at time points, pain assessment, and side effects at each time point.
(CSV)

S1 Dictionary Variable dictionary. (CSV)

## Abbreviations

IN: intranasal
INS: intranasal sufentanil
IV: intravenous
IVM: intravenous morphine
NRS: numerical pain rating scale
