# Supplementary material for: Intranasal sufentanil versus intravenous morphine for acute severe trauma pain: A double-blind randomized non-inferiority study
Source: PLoS Med. 2019 Jul 16;16(7):e1002849. doi: 10.1371/journal.pmed.1002849 (PMC6634380; doi:10.1371/journal.pmed.1002849)
Supplement: S1 Text — This file contains the complete prospective study protocol, translated from the French version approved by the regional ethics board on May 15, 2013 (Rhone-Alpes Auvergne Clinical Research Center ethics board CECIC IRB N° 2013-001665-16). (PDF) [file pmed.1002849.s002.pdf]

STUDY PROTOCOL

Title : Intra Nasal Sufentanil Versus Intravenous Morphine for Acute Severe Traumatic Pain Analgesia in Emergency Setting

Short title: ALGOFINE 2

INVESTIGATOR COORDINATOR OF THE PROJECT:

Name: BLANCHER Marc

Title or Rank: Attending physician

Contact details: SAMU 38, Emergency Department, Grenoble University Hospital, BP 217 38243 Grenoble Cedex 09. Tel: 04 76 63 02 46

PROMOTER :

Name: Grenoble University Hospital represented by the Clinical Research and Innovation Department (DRCI).

Contact details : DRCI. CHU de Grenoble BP 217. 38243 Grenoble cedex 09

Name of the person authorised to sign: Mrs Jacqueline HUBERT, CEO

ASSOCIATE INVESTIGATORS

Dr. P. Wattrelot (Grenoble University Hospital, SAU Surgery)

Dr. J. J. J. Banihachemi (Grenoble University Hospital, South Hospital)

Dr M. H. Schmidt (Grenoble University Hospital, Emergency Department / SAMU / SMUR)

Dr M. Maignan (Grenoble University Hospital, Emergency Department / SAMU / SMUR)

Dr. E. Rancurel (Grenoble University Hospital, Emergency Department / SAMU / SMUR)

Dr. R. Briot (Grenoble University Hospital, Emergency Department / SAMU / SMUR)

PRINCIPAL INVESTIGATOR OF THE ASSOCIATE CENTRE

Dr. F. X. Ageron (Annecy Hospital Centre)

PRINCIPAL INVESTIGATOR OF THE ASSOCIATE CENTRE

Dr. S. Fey (Centre hospitalier Chambéry)

PRINCIPAL INVESTIGATOR OF THE ASSOCIATE CENTRE

Dr François ALBASINI (Centre hospitalier de Saint Jean de Maurienne)

PRINCIPAL INVESTIGATOR OF THE ASSOCIATE CENTRE

Dr. Audrey WUYTS (Albertville Hospital Centre)

Others

Dr. E. Schir (pharmacovigilance, Grenoble University Hospital),

Dr. A. Lehmann (pharmacy of the University Hospital of Grenoble, Clinical Trials Department),

J.L Quesada (statistician, CHU Grenoble)

Scientific Manager of the Project

Name: Raphael BRIOT

Title or Rank: Associate Professor in Emergency Medicine

Telephone: 04 76 63 02 46

Name of Laboratory or Department: SAMU 38, Emergency Department

Name of the Laboratory Director or Head of Department: Pr Vincent Danel and Pr Françoise Carpentier

Address: Emergency Department SAMU / SMUR, Grenoble University Hospital

ABSTRACT

Introduction

In emergency medicine, patients' analgesia regularly requires the use of intravenous morphine. Obtaining an effective and rapid analgesia without systematically using an intravenous access would be an interesting alternative as it would shorten the time to achieve analgesia while using a painless and risk-free route. The use of Sufentanil by Intra Nasal route has shown its efficacy in several clinical studies but the one we propose would be the first comparative, prospective, randomized, controlled, single blind, double placebo study.

Main objective

The aim is to verify the non-inferiority of analgesia provided by Sufentanil administered by intra-nasal spray compared to intravenous Morphine in patients admitted in emergency departments with traumatic pain intensity estimated at ≥ 6 on numerical rating scale (NRS).

Secondary objective(s)

To evaluate the superiority of intranasal Sufentanil at intermediate times (10 and 20 min)
Evaluation of the tolerance of intra-nasal Sufentanil: description of adverse events.
Feasibility of intranasal Sufentanil analgesia in the prehospital setting

Main judgment criteria

Non-inferiority of analgesia at 30 min: comparison of the variation in the numerical pain scale (NRS at the beginning of analgesia - NRS 30 min later), between the reference treatment (Morphine IV) and the tested treatment (Sufentanil IN).

Secondary judgment criteria

Efficacy of analgesia at intermediate times (10 and 20 min).
Occurrence of adverse events.
Efficacy of analgesia at times (10, 20 and 30 min) in the pre-hospital patient group.

Target population (total number of subjects)

All patients presenting to the emergency department or managed by a prehospital mobile intensive care unit with self-assessed traumatic pain intensity ≥ 6 on the numerical rating scale.
218 participants, 109 in each arm for the main criterion. The assessment of pain in the pre-hospital patient group, corresponding to a secondary objective, requires 30 additional subjects.
Study duration by subject: 4 hours
Total duration of the study: 36 months

Methodology and methodology

Randomized, single-blind, double-blind, placebo-controlled, multicenter study.

Each patient receives at each step of the following:

- Intravenous injection of Morphine (10 mg/mL) or placebo (sodium chloride 0.9%)

And

- One to two intra-nasal sprays of placebo or Sufentanil (50µg / ml)

Data analysis

The statistical analysis of the data will be carried out at the Grenoble University Hospital by Dr Jean Louis Quesada, Biostatistician (Clinical Research and Innovation Department - DRCI), in collaboration with Dr Marc Blancher.

Main objective: the non-inferiority of the treatment evaluated compared to the reference treatment in terms of NRS at 30 minutes (variation of NRS Pre-After analgesia) will be evaluated using the Schuirmann test[26] (one-sided mean-equivalence t test (two one-sided t tests approach, TOSTT procedure, per protocol analysis). The non-inferiority threshold used for this analysis will be 1.3. The statistical significance threshold will be 2.5%.

As regards the analysis of the occurrence of side effects and, in general, all qualitative parameters, they will be expressed in numbers and percentages and will be analysed by the chi2 test or the exact Fisher test, the significance threshold being set at 5%.

Concerning the analysis of the effectiveness of analgesia at intermediate times (10 and 20 minutes) and generally all quantitative parameters, they will be summarized by the mean and standard deviation when normality is confirmed, otherwise they will be expressed as median and the 25th and 75th percentiles. Inter-group comparisons of these parameters will be made using the Student or Mann-Whitney tests. The significance threshold is set at 5%.

The overall descriptive analysis will be carried out on the population (intention to treat).

Expected results:

We expect to show the non-inferiority of intra-nasal Sufentanil analgesia compared to intravenous morphine for trauma patients experiencing severe pain in the emergency department. This study could pave the way for more frequent use of intra-nasal analgesia in emergency medicine. It appears safer (in terms of infectious risks), faster and less painful than the intravenous route. The realization of such an effective and rapid analgesia by non-invasive means could then be routinely considered in emergency departments, to reduce the time required to relieve pain of trauma patients, or in situations where the insertion of a peripheral venous catheter is particularly delicate or even impossible, particularly in the prehospital setting.

SUMMARY

I. - Rationale for the study: 6

II. - Objectives of the study: 11

III. - Experimental design 12

IV. - Characteristics of the participants 17

V. - Technical data on experimental equipment or drugs 19

VI. - Measured variables and measurement methods : 25

VII - Data collection and management: observation booklet - quality control 26

VIII. - Sample Collections: 26

IX. - Statistical analyses : 26

X. - Calendar: 30

XI. - Material and legal aspects: 30

XII- Adverse event(s) : 33

XIII - Early termination of research: 35

XIV - Archiving: 36

XV - Publication : 36

XVI - Bibliographical references: 38

I. - Rationale for the study:

Analgesia is one of the main objectives of the management of patients presenting themselves in an emergency department (ED). Trauma is more particularly associated with acute painful episodes, this is the case at the time of the trauma but also when performing procedures in the ED such as removal of clothing, clinical or radiological examination. The Grenoble area and the Alpine region have the dual characteristic of being both very dense in terms of population and geographically located very close to mountain areas. As a result, in addition to the "classic" sports and domestic trauma, trauma related to mountain sports, and in particular winter sports, are frequent. While the pain is particularly associated with trauma, it is also very often underestimated, which can have deleterious consequences on the clinical condition of patients. Reducing trauma pain is one of the public health objectives that has been clearly defined by the Ministry of Health.

Pain intensity is assessed using self-assessment techniques, in particular the 11-point numerical rating scale (NRS) from 0 (no pain) to 10 (maximum pain imaginable). Pain is considered severe when it is rated 6[1] or higher. For an adult patient, the standard treatment for severe pain in the ED is intravenous morphine as follows: either 2 mg (< 60 kg) to 3 mg (>60 kg) injections every five minutes; or as a bolus from 0.05 to 0.1 mg/ kg, then reinjection of 2 to 3 mg (0.025 to 0.05 mg/ kg) every 5 minutes until a pain self-assessment score is obtained ≤ 3 on NRS[2].

Other products derived from Morphine are usable and have a theoretical interest because they act faster. This is the case with Sufentanil, which has a shorter onset of action (60 to 120 seconds) than Morphine[3]. In addition, Sufentanil has the advantage of a wider therapeutic range than Morphine or Fentanyl. In practice, studies have shown few adverse effects when using Sufentanil[4-6].

The intravenous route of administration is the reference in analgesia for severe pain, but it is carried out only at the cost of a painful and anxiogenic act since it requires a venous puncture and catheterization. In addition, not all trauma patients require the maintenance of a venous access after prior analgesia in the ED. However, it is important to maintain the possibility of rapid and effective analgesia in the event of acute pain: intra-nasal Sufentanil would be perfectly suited to this situation.

It is therefore desirable to have a non-invasive, reliable and safe morphine delivery route. This route must make it possible to obtain analgesia in a short period of time, which implies the use of products with a short duration of action. The oral access does not appear to be the best option since the time to act is long.[1] The rectal access is also not an option for practical reasons and above all because bioavailability varies greatly from one subject to another. While the transmucosal pathway (sublingual and endobuccal) has proven its effectiveness, it has never been studied in the emergency setting. In addition, the onset of action of morphine derivatives is long and patients are not particularly satisfied with this mode of administration [7]. The intra-nasal route appears to be an interesting and promising access. It has been the subject of numerous studies

comparing its efficacy and safety in painful postoperative situations [8-10], for dental care [11], for burns care [12] and especially in pre-hospital [13] or hospital emergency situations [14, 15, 16].

Physiological specificities of the intra-nasal route:

The intra-nasal cavity consists of several types of mucous membranes. The most important one, the respiratory mucosa, offers an absorption surface area of 150 to 160 cm^2 allowing active products to pass through the bloodstream[17]. The vestibule and atrium areas are very small and have no absorption capacity, unlike the olfactory mucosa area with a surface area of 6 to 10 cm^2 located in the upper part of the cavity, which allows direct crossing of the hemato-encephalic barrier (via the passage of the sieve blade)[18].

Via this mode of intra-nasal diffusion, there is no intestinal absorption of the products and therefore no effect of first hepatic passage, unless the sprayed volumes are too large. The maximum spray dose to avoid passage through the digestive tract is 0.15 ml.[10] This is an essential element, as this very limited volume requires the use of high concentrations, which are all too rarely marketed. As local blood flow rates are very high in the nasal mucous membranes, the bioavailability of the spray products is very good, especially if they are liposoluble. For Sufentanil, bioavailability is 78%[4].

Clinical studies concerning intra-nasal analgesia:

Studies published to date tend to show the efficacy and safety of intranasal Sufentanil in the management of pain in the ED. However, there are no randomized, controlled, blind studies conducted in ED in an adult population.

The comparison of intranasal Sufentanil IN to intravenous Sufentanil in a small group of patients showed a very promising efficacy and tolerance of the intranasal pathway for this molecule.[4] A study was published in 2012, involving 40 patients with acute trauma pain relieved by a dose of 0.5 µg / kg of intranasal Sufentanil.[5] This dose is more than 4 times higher than the analgesic potent of intravenous morphine. Pain was assessed before administration of intransal Sufentanil, 10, 20 and 30 minutes later by the numerical scale. It should be noted that for an average administered dose of 37.7 µg per patient, pain was reduced by 4.7 points on a digital scale from 10 min and by 5.7 points at 20 min and 30 min. The adverse reactions were rare, the most frequent were dizziness (3 patients or 7.5%) and oligoanalgesia (2 patients or 5%). One case (2.5%) of vomiting and one case of hypoxemia (SpO2 <88%) were reported. No hypotension or apnea was detected. It can be noted that the volumes used are often much higher than the maximum optimal absorption volume of 0.15 ml per nostril. For example, for patients over 96 kg, the volume used was 0.55ml per nostril. This is more than three times the theoretical maximum absorption volume of the nasal mucosa, yet there have been no reports of decreased analgesic efficacy in this population. These data are therefore reassuring regarding this theoretical limit of 0.15ml per nostril, which seems to be

exceeded without risk or loss of effectiveness. Overall satisfaction with this protocol was 78% among patients and caregivers. Other studies [6,19,20] have used intranasal Sufentanil for analgesic purposes or as a pre-induction of anesthesia in different populations (adults, children, young children) and have not reported serious or unusual adverse events at doses less than or equal to 0.5 µg / kg.

A larger number of studies using intranasal Fentanyl, a molecule similar to Sufentanil, are available. However, only low concentration of Fentanyl are available making its use almost impossible in the emergency setting. An Australian study published in 2007, on a population of children in the ED showed that there was no difference between the analgesia performed by intravenous morphine and by intranasal Fentanyl. There were no more side effects in the intranasal arm than in the intravenous arm.[15] On the other hand, a retrospective study carried out by the same team found that the use of the intranasal route allows a quicker relief of pain thanks to the simplicity of this route of administration.[14] This was confirmed by Holdgate's study which showed that the time to receive analgesia decreased from 60 to 30 minutes when the intranasal route was used instead of the intravenous route.[13] Another study highlighted the fact that the use of intranasal fentanyl in pediatric emergencies reduced the occurrence of catheter obstruction from 100% to 42%. This improved the quality of care for children and reduces the workload for emergency staff.[21] Rickard's Australian study in pre-hospital emergency medicine also showed that the efficacy of analgesia was not different between intranasal Fentanyl and intravenous Morphine without any further side effects [22].

<u>Problem of concentration of intra-nasal opioids:</u>

In Australian studies, intranasal Fentanyl concentration was 300 micrograms/ml, sprayed via an intranasal cap system including a Mucosal Atomization Device. However, this dosage is not available in daily practice. Indeed, Fentanyl vials are marketed at a dose of 50 micrograms per ml. This dosage would involve spraying doses that would actually exceed the maximum theoretical absorption capacity of 0.15 ml / nostril. This is why the use of Sufentanil, marketed at a concentration of 50 µg / ml, seems to be a good alternative. This concentration allows a lower volume delivery, therefore with less digestive absorption and better control of analgesia.

<u>Sufentanil MYLAN 50 µg / ml injectable solution (IV and Peridural) :</u>

This product, generic drug of the SUFENTA® referent, is indicated as an adjunct analgesic treatment during general anesthesia; or as an epidural analgesic; or as a main anesthetic during analgesic anesthesia; or as a sedation agent for ventilated patients.

The product is available in a concentration of 50 µg /ml, sufficient for intra-nasal use, which requires reduced volumes.

It is a very fat-soluble molecule. It binds to plasma proteins for 90 to 92.5% of the dose. The pKa is 8.01, which corresponds to an ionized fraction of 80% at pH 7.40. This ionized fraction varies with pH, but in small proportions, less than Fentanyl. Sufentanil is mainly eliminated after metabolization, carried out in the liver (by cytochrome P450 3A4) and small intestine. The intransal route avoids this first-pass hepatic effect, allowing precise dosage adjustment. Within 24 hours, 80% of the dose is eliminated, including 2% in unchanged form.[23] Adverse reactions are those of opioids, the most serious being ventilatory depression, apnea, muscle stiffness (especially chest stiffness), myoclonic movements, bradycardia, hypotension (transient), nausea or vomiting and dizziness. Other less frequently reported adverse events are: laryngospasm, Sedation, pruritus, nausea and/or vomiting, respiratory depression with bradypnea and/or apnea can be observed after epidural or intrathecal administration. An overdose of Sufentanil results in an exacerbation of pharmacological signs. Ventilatory depression is the main clinical sign and varies, depending on individual sensitivity, from bradypnea to apnea.

<u>Problem of the analgesic dose and the dose to be tested:</u>

The theoretical equivalent analgesic dose of intravenous Morphine corresponds to an intransal dose of Sufentanil of 0.14 µg / kg. Indeed, Sufentanil is 1000 times more powerful than Morphine. Therefore, 0.1 mg / kg of Morphine IV is equivalent to 0.1 µg / kg of Sufentanil IV. In addition, the bioavailability of Sufentanil by the intranasal route is 78% [4]. Other data on the bioavailability of intranasal morphine are for Fentanyl and consider that 1 mg of Morphine IV would correspond to 1 µg of Fentanyl IV and 1.4 µg of Fentanyl IN.[17,24,25] Sufentanil being 10 times more concentrated than Fentanyl, we deduce that :

0.1 mg / kg Morphine IV = 0.1 µg / kg Sufentanil IV = 0.14 µg / kg Sufentanil IN.

However, the data in the literature tend to show that the dose with the best efficacy / tolerance ratio is above this theoretical dose:

- A post-operative pain study used repeated bolus every 5 minutes as often as necessary, of 0.025 µg / kg (mean total dose of 0.18 µg / kg for the first hour) vs 0.05 µg / kg (mean total dose of 0.24 µg / kg for the first hour). This last higher dose proved to be more effective without any risk. The average total dose of Sufentanil delivered in the first hour was 0.24 µg/kg [19]
- A dose of 0.5 µg / kg is used in a 2012 emergency medicine study in a group of 15 subjects, and appears to be effective and well tolerated [6].
- This same dose was successfully used in 2012 in a group of 40 patients with acute trauma pain. The average dose used was 37.7 µg per subject, with doses up to 40.1µg [5].

- The use of intransal Sufentanil at doses up to 1.5 µg / kg does not present any risk of serious adverse reactions. Doses up to 4.5 µg / kg have been tested, but does not present an interesting benefit/risk ratio for analgesic use.[20]

Facing the difference between theoretical (0.14 µg / kg) and empirical (0.5 µg / kg) data, we chose an intermediate dose of 0.3 µg / kg, allowing, based on the literature review, to propose an effective analgesia for severe pain while ensuring a completely satisfactory safety margin.

Concerning the administration modalities: Is it necessary to have a single dose in a single nostril? Half a dose in each nostril? Or several repeated sprays?
There are no data in the literature about intranasal Sufentanil. On the other hand, there are some on Instanyl® (Fentanyl), used by the intransal route. Pharmacokinetic studies used to support the European Medicines Agency (EMA) approval,[14] show that efficacy decreases if several successive sprays (less than 5 minutes apart) are carried out in the same nostril. Two doses of 50 micrograms in the same nostril would therefore be less effective than a single dose of 100 micrograms. These data concerning a molecule close to Sufentanil (although less lipophilic) may be useful for the drafting of the study protocol.

Conclusions :
For the intranasal route, Sufentanil dosed at 50 micrograms per millilitre appears to be a product suitable for emergency analgesia in the context of acute trauma pain in adult naïve morphine patients. There would be no more side effects from this route than from the intravenous route with possible same analgesic efficacy. However, this hypothesis must be tested in an adult population in ED suffering from acute trauma pain by a randomized/controlled/blind study in order to obtain a sufficient level of evidence to recommend its routine use. The optimal dose to be tested is 0.3 µg / kg based on analysis of literature data.

II. - Objectives of the study:

Main objective:

To verify the non-inferiority of analgesia provided by Sufentanil administered by intra-nasal spray compared to intravenous Morphine in patients admitted in emergency departments with traumatic pain intensity estimated at ≥ 6 on numerical rating scale (NRS).

Secondary objectives:

To evaluate the efficacy of intranasal Sufentanil at intermediate times (10 and 20 min)

Evaluation of the tolerance of intra-nasal Sufentanil: description of adverse events.

Feasibility and efficacy of intranasal Sufentanil analgesia in the prehospital setting

Main judgment criterion:

Non-inferiority of analgesia at 30 min: comparison of the variation in the numerical pain scale (NRS at the beginning of analgesia - NRS 30 min later), between the reference treatment (Morphine IV) and the tested treatment (Sufentanil IN).

Secondary criteria :

Comparative efficacy of analgesia after 10 and 20 min: comparison of the variation in the numerical pain scale (NRS initial - NRS at 10, NRS at 20 min) between the reference treatment (Morphine IV) and the tested treatment (Sufentanil IN).

Occurrence of adverse effects defined as: Nausea, vomiting, epistaxis, nasal discomfort, deep sedation (sedation scale > 2), desaturation < 90%, blood pressure < 90 mm Hg, respiratory < 10 / min, use of Naloxone (antidote).

Comparative efficacy of analgesia after 10, 20 and 30 min in the prehospital setting: comparison of the variation in the numerical pain scale (NRS initial - NRS at 10, NRS at 20 min) between the reference treatment (Morphine IV) and the tested treatment (Sufentanil IN).

III. - Experimental design

A- type of search: clinical research

B- Purpose of the research: pharmaceutical

C- Nature of the Research: phase III

D- Multicentric Project: yes

E- Multidisciplinary project: no

F- Randomization: yes

Rationale: Our study compares two groups of patients receiving two different treatments.

Method: Randomization is carried out via an intranet site of the Grenoble University Hospital by blocks of random sizes and stratified by centre.

The randomization list is given to the central pharmacy of the University Hospital which prepares all the analgesia kits.

G- Blinding methods: yes

This is a single-blind, double-blind, placebo-controlled study. Subjects do not know in which arm they are assigned. They will receive either Morphine IV + placebo IN or Sufentanil IN + placebo IV. The products used for the study are packaged in dedicated study kits. The kits are perfectly identical to each other before opening. They are manufactured at the central pharmacy of the Grenoble University Hospital in the clinical trials department, which will have the randomization list. The investigator only discovers the arm assigned to the patient after the inclusion and the randomization. The assignment of the treatment arm remains secret and the investigator does not have the possibility to choose in advance. This method appears to be the most suitable for emergency situations because it is available at any time, even in the absence of a telephone network in the pre-hospital phase, unlike centralized randomization.

Each kit contains a randomization number (which is also the kit number), the necessary equipment to perform analgesia and placebo. The allocation of kits per patient is done in chronological order of the randomization numbers. The removal of the blind may be carried out at any time by the principal investigator in the event of a serious and unforeseeable adverse event.

H- General organization of the study:

The study is conducted either in the adult ED or in the prehospital setting in mobile intensive care units (MICU). All persons involved in the study (physicians, nurses) will have previously received specific training on the conduct of the study and on the administration of Sufentanil by the intranasal route. Inclusion is allowed 24/24h. In the ED, during weekdays and during working hours, a team will be specifically dedicated to the inclusion of patients.

Subjects with inclusion criteria (trauma pain, self-assessed intensity by NRS ≥ 6) will be identified by the triage nurse or by the team of the MICU. There will be no change in the care of the patients, except for analgesia. With regard to analgesia, the clinical investigation team will assess whether there are any criteria for non-inclusion. In the absence of criteria for non-inclusion, specific information will be given orally and in writing to the patient who will be required to sign a consent form. If the intensity of pain do not allow the patient to complete and sign the consent form, the consent may be signed by a representative. In cases where the patient's condition and the absence of family members or trusted person designated by the patient do not currently allow them to receive information regarding the clinical study, and pending the possibility of obtaining a consent signed by the patient or a family member or trusted person designated by the patient after the completion of the study, the principal investigator or associated investigators may include the patient in this study. In the event of a subsequent refusal of the patient's consent, all data concerning the patient will be destroyed. If all these conditions are met, the patient will enter the study. Kits will be assigned in ascending order of randomization number (which will also be the kit number) according to the order of arrival of the patients, the randomization having been previously performed. If the investigation takes place in the ED, the patient will be placed in an examination room. An emergency physician will carry out the usual clinical examination and will prescribe the necessary additional examinations. The analgesia will be performed by the investigating team. If the patient is managed by a MICU in the prehospital setting, the clinical examination will be carried out normally and analgesia will be started in the prehospital.

Preconditioning :

The patient's weight will be estimated. A peripheral venous access will be placed and connected to a normal saline infusion. Continuous monitoring of heart rate, non-invasive blood pressure and oxygen saturation will be implemented.

Each patient will have an inclusion number and an analgesia kit designed by the pharmacy. This kit consists of an active product and a placebo. Under no circumstances the patient will be in a position to know which of the IV or IN product is the active product and which is the placebo. Randomization will be performed *a priori* before the kits are manufactured. Removal of the blindness will only be done by the principal investigator at the end of the study or in case of a life threatening adverse event.

Preparation of experimental drugs:

Once opened, the kit will have 2 clearly differentiated zones: one containing the identified material: track IV, and the other identified: IN track.

Morphine or placebo IV

The preparation of the IV track material will be carried out in a standard way: the needle is inserted at the end of the 20 ml syringe, two vials of Morphine Aguettant 10 mg/ml and the 0.9% NaCl bottle of 20ml or only the 0.9% NaCl bottle of 20 ml will be opened. Out of sight of the patient and the physician in charge, the syringe will be filled with 2 ml of Morphine and 18 ml of 0.9% NaCl (for a morphine concentration of 1 mg/mL) or 20 ml of 0.9% NaCl. Finally, the needle is removed, the syringe is connected to the IV line and the corresponding volume is injected.

The part concerning the filling of the syringe with Morphine/Placebo will be done out of sight of the patient and the physician because the vials differ. In this way, it is impossible for the patient and the physician to differentiate Morphine from Placebo.

Sufentanil or normal saline by IN route

The preparation of the IN track material is specific. Indeed, in order for IN spraying to be as effective as possible, it is important to push the piston vigorously. However, to respect the accuracy of the doses administered, it is necessary to fill the 1ml syringe only with the dose to be administered (taking into account the dead space of the intra-nasal device, detailed below). The steps of the realization will therefore be as follows: the needle will be screwed onto the 1 ml syringe, the vial of Sufentanil Mylan 50 µg / mL (5 ml vial) or the 0.9% NaCl vial of 20 ml will be opened, the syringe will be filled with the volume corresponding to the patient's weight to which at least 0.1ml must be added to "purge" the dead volume of the intranasal device (refer to the table in Appendix 10). The needle will be removed, the Mucosal Atomization Device (MAD) is screwed to the end of the syringe, the piston of the syringe is pushed in order to adjust the volume to be administered (this allows the intra-nasal device to be purged), the corresponding volume is administered by spraying into a nostril.

One element to consider is the technical aspect of the MAD. The nasal tip has a "dead space" of 0.1 ml, which is not negligible considering the volumes administered. Indeed during the administration this volume will remain in the device. The instructions for use of the MAD recommend taking an additional 0.1 ml from the syringe to compensate for this "dead volume". For a perfect adjustment of the doses we therefore propose to take 0.1 ml more than the volume to be administered with the syringe and to carry out a "purge" of the intra-nasal device just before administration. This means that the user of the device will push the piston of the syringe (intranasal device) until the exact volume to be sprayed is obtained in the syringe, so that a precise dose can

be administered. In this way the dead volume of the device will be filled with product, so there will be no loss of volume at the time of administration.

Analgesia will be started as follows:
Intravenous (IV) Intravenous
From the 20 ml syringe of the Kit, an IV injection of **0.1 ml / Kg** body weight is performed.

Intra-nasal route (IN)
At the same time as the IV injection, two intra-nasal sprays are performed (see dose / weight table in Appendix 2), corresponding to a dose of 0.3 µg / kg. The patient is in supine position at 30°.

Vital parameters will be continuously monitored and collected in the observation logbook every 10 minutes as well as the self-assessed intensity of pain (rated from 0 to 10 on the NRS).

If the NRS is > 3 after 10 min, a reinjection of 0.05 ml / kg of IV product **and** an IN product spray will be performed. This corresponds to an active product dose of 0.05 mg / kg Morphine or 0.15 µg / kg Sufentanil. At any time, the investigator will note the subjective side effects reported by the patient (nausea, vomiting, bad taste, irritation, epistaxis)...
Analgesia will be considered sufficient and the patient relieved if the NRS is ≤ 3 within 30 minutes of the start of the analgesia. After 30 minutes after the first injection, if the patient is still not relieved (NRS>3), another analgesic strategy will be considered (rescue strategy).
Coanalgesia with products of level 1 or 2 will be possible according to the service protocols.

In case of signs of severe morphine overdose, (respiratory rate <10 / min or sedation score > 2), opioids will be immediately stopped, the patient will be verbally stimulated by the investigator and oxygen therapy will be started. If these measures are insufficient, the investigator will inject Naloxone intravenously according to the following protocol: a 0.4 mg / ml vial is diluted in a 10 ml syringe (9 ml of 0.9 NaCl + 1 ml of Naloxone). The injected dose is 1 ml (0.04 mg) to be repeated every 60 seconds until respiratory rate >12 / min.

I- Procedure conducted and differences from usual care

Only the use of two simultaneous routes of administration changes the usual management.

J- Study locations

ED and MICU of the Grenoble University Hospital (north and south hospitals)
ED and MICU of the Annecy Regional Hospital Centre,
ED and MICU of the Chambéry Hospital Centre,
ED and MICU of the Saint Jean de Maurienne Hospital Centre

ED and MICU of the Albertville Hospital Centre

IV. - Characteristics of the participants

Sample size of the study

This study will involve 198 patients (99 patients per group). We plan to recruit an additional 10 patients per group to account for lost of follow up and withdrawal. The calculation of the number of patients required is based on a **non-inferiority study** design.

The null hypothesis corresponds to a difference in efficacy between treatment groups greater than the equivalence threshold. The alternative hypothesis makes it possible to conclude that the treatments are equivalent. The main criterion is the difference in the mean pain score evaluated by NRS at inclusion and 30 minutes after administration (after minus before).

The number of subjects required was evaluated by the Schuirmann method,[26] taking into account a minimum, clinically significant value of change in pain intensity defined a priori as a 1.3 change io the NRS,[24] with an alpha risk of 2.5% and a standard deviation of the response of 2.8.[27] A number of 99 subjects per group will thus be necessary to reject the null hypothesis of a difference in efficacy and conclude that the treatment evaluated is not inferior to the reference treatment, in terms of the evolution of NRS in the first 30 minutes, with a power of 80%. This calculation was performed using the NQuery Advisor software version 7.0 by the scientific department of the DRCI (Biostatistician JL Quesada).

The assessment of feasibility (pain and satisfaction) in the pre-hospital patient group, corresponding to a secondary objective, will be carried out on a population of 30 subjects, set to provide an initial descriptive assessment.

Source of recruitment of subjects

Patients presenting themselves to the ED of the study centers and patients treated by a Mobile Intensive Care Unit of these same centers.

Inclusion criteria

Patients presenting to the ED with pain due to trauma and self-reporting a pain intensity ≥ 6 on NRS. Pain intensity can be either permanent or paroxysmal or triggered by movement or mobilization.

Age between 18 and 75 years old

Patient affiliated to social security or equivalent

Informed and written consent signed by the patient or his or her legal representative.

Criteria for non-inclusion

Age < 18 years or age > 75 years

Multiple (suspected or not) trauma patient

Medical pain

History of chronic respiratory, renal or hepatic insufficiency

Any drug addiction

Pregnant woman or breastfeeding woman.

History of surgery of the nose and/or chronic nasal pathology (including sinuses)

Transcutaneous pulsed saturation of oxygen < 90%

PAS < 90 mmHg

Opioid allergy

Head injury with neurological impairment (GCS Score < 14)

Massive facial trauma

Patient unable to understand or rate the intensity of pain on a NRS

Patient who received a morphine-type analgesic (level 3) within 6 hours prior to management

Loco regional anesthesia indication

Obstruction of the nasal passages treated with nasal decongestants (especially oxymetazoline) within 20 minutes before admission.

Protected persons referred to in Articles L1121-5 to L1121-8 of the French Law

Uncontrolled epilepsy

Intracranial hypertension

Bodyweight >100kg

Exclusion criteria

Violation of protocol.

Authorized / prohibited processes

The use of drugs derived from morphine and nasal decongestants, in particular oxymetazoline is not compatible with this protocol.

Exclusion period

A 24-hour exclusion period after the study and a prohibition to participate in other research simultaneously are provided for.

Financial compensation

None

Registration in the volunteer database

No registration in the volunteer database is planned.

V.- Technical data on experimental equipment or drugs

All the medicines used are approved by both the European and the French Health Authorities. The Mucosal Atomization Device is also approved by these authorities. The product supply will be provided by the Pharmacy of the University Hospital of Grenoble.

Morphine IV is morphine hydrochloride used routinely, dosed at 10mg/ml and marketed by the Laboratory AGETANT.
Sufentanil IN is sufentanil citrate at 50 µg/ml. It is marketed by the Laboratory MYLAN as an injectable solution (IV or epidural), packaged in 5 ml vials each containing 250 µg of Sufentanil. The excipients present in this form are water for injection, sodium chloride, sodium hydroxide and hydrochloric acid, compatible with intra-nasal administration.

Summaries of Product Characteristics and operating instructions are available in Appendix 4.
The Mucosal Atomization Device used in this study is marketed by Wolfe Tory Medical, 79 West 4500 South, Suite 18, Salt Lake City, UT 84107, USA (see Appendix 5).

A- Securing the morphine circuit

a. Preparation of the kits

The kits will be prepared with the help of the pharmacotechnic laboratory of the University Hospital of Grenoble (clinical trials department).
The active products and placebos required for the study will be stored in a secure safe in the clinical trials department of the central pharmacy of the Grenoble University Hospital before being packaged in a kit.
All kits will be manufactured over a short period of time in order to limit the risk of error associated with "on-demand" manufacturing.
Given the space available to store the prepared kits, it is possible to plan the production of 100 kits stored at the central pharmacy of the University Hospital of Grenoble, and 20-30 kits for each other center (Chambéry, Annecy, Hôpital Sud de Grenoble, Saint Jean de Maurienne and Albertville). An additional 30 kits are planned for the secondary objective for pre-hospital patients.

Once sent to the different centres of inclusion, the 30 kits will be stored in the pharmacy vault at each centre. Each pharmacy provides kits to investigators according to the frequency of inclusions, with a secure safe reserved for the ALGOFINE 2 study being made available to each investigator service.

b. Contents of the kits

For all kits:

1 cardboard box

1 foam block with space for the material

1 label on the outside of the lid with the randomization number, the name of the study, the name of the investigator coordinating the project and the name of the study sponsor, which seals the opening of the box

1 label on the inside of the lid of the box containing the subject's randomization arm, the course of analgesia and the doses to be administered.

Paper documents:

1 study procedure (APPENDIX 7)

1 dose table according to weight (APPENDIX 10)

1 drug administration record (APPENDIX 12)

1 Serious Adverse Event Reporting Form (APPENDIX 8)

For Morphine IV group (reference arm): red color code

A 20 ml syringe, an 18G needle 1½" of 1.2 x 40mm, a 20 ml vial of 0.9% NaCl, two vials of 1ml Morphine dosed at 10 mg / ml, all identified: "IV route only".

An intra-nasal device, a 1 ml screw syringe, an 18G needle 1½" 1.2 x 40mm, a 10 ml vial of 0.9% NaCl and all identified as "Intra-Nasal only".

For the Sufentanil IN group (experimental arm): blue color code

A 20 ml syringe, an 18G needle 1½" 1.2 x 40mm, a 0.9% NaCl vial of 20 ml, all identified as "IV only".

An intra-nasal device, a 1 ml screw syringe, an 18G needle 1½" of 1.2 x 40mm, a 5 mL vial of Sufentanil dosed at 50 µg / ml and all identified: "Intra Nasal route only".

c. Securing the morphine circuit

Given the products used and the multicentric nature of this study, the safety of the morphine circuit from their control to their destruction via their administration is a priority. The means put in place to limit incidents are multiple:

- Designation and training of one pharmacist and one investigator per centre responsible for the proper conduct of the study
- Opioids are stored as soon as they are received in the vault of the pharmacy of the Grenoble University Hospital

- All products (except needles, identical between IV and IN) as well as Morphine and Sufentanil are labelled to avoid any confusion (see the following chapter "Labels")
- The syringes used to prepare and administer the products are totally different: it is a 1ml screw syringe for the IN route, and it is a 20ml screwless syringe for the IV route.
- In the kit, all the material is stored in slots cut out of a foam block. These are divided into 2 zones delimited by the usual colour code (red for track IV and blue for track IN), limiting any errors when preparing the syringes.
- The location of materials and drug substances in the kit is carried out by a person trained for this specific task. A verification of this arrangement is carried out by a second trained person. In addition, the locations are cut to the exact size of the corresponding pharmaceutical products and devices.
- The randomization number is written on the kit, allowing traceability despite the closing of the kit.
- The kits are sealed with an indelible label, making any opening permanent and visible in an obvious way.
- All the kits are produced before the start of the study, over a short period of time.
- The kits are sent via authorized carriers to pharmacies in the centres of the study
- Each centre stores the kits (20-30 per centre and 6-8 additional kits for the pre-hospital group) in the safes or vaults of the pharmacy.
- A secure safe will be installed in each ED participating in the inclusions, allowing one kit to be immediately available. The pharmacy of each centre will replace the kits in those safe, which are managed locally by the department manager, the pharmacist in charge of the centre and the principal investigator of the centre.
- When the kit is opened, the nurse checks and validates the presence of the drugs corresponding to the group specified in the kit, as well as the correct dosage (label to be completed by the nurse and attached to the administration record)
- The preparation and administration of products (NaCl, Morphine or Sufentanil) by the IN or IV route under this protocol is a medical act and cannot be delegated.
- All equipment (documents, empty bulbs, unused bulbs) is put back into the kit after use.
- The kits used or violated are stored in the lower floor of the secure cabinet.
- The kits used, violated or not used at the end of the study are sent via an authorized carrier to the pharmacy of the Grenoble University Hospital.
- The verification and destruction of opioids are carried out at Grenoble University Hospital.
- The clinical research assistant in charge of the project is responsible for verifying the smooth running of this circuit by making regular visits to each centre.

d. Labels (ANNEX 10)

Product identification labels will be affixed to the various products to avoid any possible confusion between the two routes of administration. Each label contains:

- The color code (Red for channel IV and Blue for channel IN)
- The route of administration recalled in any letter: *"Intravenous route only"* or *"Intra Nasal route only".*
- The name of the protocol: *"ALGOFINE 2 study"*

Opioids identification labels will be fixed directly to the glass vials to avoid any possible confusion.

The label validating the presence of morphine at the correct dosage by the nurses will be fixed to the medication administration record (pink sheet, Appendix 12), once validated by the nurse. It makes it possible to verify of the presence of opioids vials:

- one 5mL vial of sufentanil (250µg-5mL or 50µg/mL) for the SUFENTANIL group (checkbox)
- TWO 1mL vials of morphine (10mg - 1mL or 10mg/mL) for the MORPHINE group (checkbox)

This label states:

- The randomization number (= kit number)
- The date of inclusion
- The nurse's initials

B- Preparation and treatment administration follow-up sheets

Traceability sheet for the production of kits (APPENDIX 13): the pharmacy produces the kits according to randomization and stores the production sheet describing the exact content (randomization number (which is also the kit number) / supplier / expiry date of drugs). This sheet allows to trace the manufacture (date, name of the person performing, name of the person checking)

The Treatment Exemption Order (APPENDIX 11) allows for the monitoring of the exemption. This form will be kept in the ALGOFINE 2 study treatment storage safe until the end of the study treatment. At the end of this period, this form will then be inserted into the file of treatment administration forms located in the patient's observation booklet to ensure the traceability of the preparation.

The processing administration form (APPENDIX 12) corresponds to the traceability sheet for the administration of narcotic drugs. It includes the date and time of administration, the investigator centre, the patient's initials, the volumes of injected and sprayed doses, the name of the prescriber and the person who administered the products.

C- Storage of study drugs and study materials

The drugs and equipment (packaging, labels and preparation tracking sheets) required for the study will be stored in the pharmacy in the vault, on shelves dedicated to the ALGOFINE 2 protocol.

a. Summary of hospital pharmacy activity:

The pharmacy will carry out in collaboration with the team in charge of the study:

- Order of the necessary consumables and drug materials.
- Assembly of kits, fixing labels on the box, on consumables, on drug vials, as well as on the drug administration record sheet.
- Storage of the various consumables and drug vials in their intended places (cut out of a foam block at the size of the cardboard box) in each kit.
- Sealing of the kit by an indelible sticker.
- Enter the corresponding randomization number.
- Traceability of the dispensing of kits in the different centres. Recovery of used, violated and unused kits
- Destruction of spared opioids

b. Pharmacy circuit: kit management

The treatment kits will be sent by the Grenoble University Hospital pharmacy to the pharmacies of the investigator sites by authorized carrier. The kits will be sent in a group, in one go, to the associated centres. The return will be done in the same way, in one go. Jean-Louis Quesada will provide 6 sealed envelopes containing the randomization lists of each centre. Each list will be sent with the kits.

Conditioning

250 kits are packaged at the central pharmacy of the Grenoble University Hospital, Clinical Trials Department. 10% of spare kits are provided for. The kits will be sealed at the end of each patient visit by a nurse.

It was agreed that the kits could be unsealed during monitoring visits then resealed at the end of the visit with a new label and a traceability sheet indicating the monitoring date and the signatures of the present people.

Dispensation of products to centers

Each pharmacy in each centre will provide the investigators with the kits planned for the study, in numbers of 5.

Labelling

All consumables (except needles), as well as vials of morphine and sufentanil are labelled to avoid confusion.

Storage

The drugs and equipment (packaging, labels and preparation tracking sheets) required for the study will be stored in the pharmacy in the vault, on shelves dedicated to the ALGOFINE 2 protocol. Once sent to the centres, the kits are stored in the pharmacy vault of each centre. Each pharmacy provides emergency services in kits according to the frequency of inclusions, with a secure safe reserved for the ALGOFINE 2 study being made available to each ED.

Shipping

The shipping and return of the kits from the 6 centres will be handled by the carrier CETUP. Delivery and reception times will be scheduled between Grenoble University Hospital and the associated centre in order to ensure the presence of the pharmacy to receive the kits.

Traceability/ Control

It is planned:

- a form to trace the double-checking of the distribution of materials and drug substances (date, name of the person performing, name of the person checking),
- a treatment delivery order form to track the preparation and follow-up of the dispensation at the centres,
- an administration record sheet tracking the administration of narcotic drugs.

The randomization number on the kit allows traceability despite the closing of the kit.

All the material (documents, empty vials, unused vials) is placed back in the kit at the end of the visits in order to ensure the traceability of the entire inclusion process.

Accounting and return

During the study, centralization is done at the pharmacy of the centre.

At the end of the study, the centres must return all used, violated or unused kits to the pharmacy of the Grenoble University Hospital.

A verification of the consistency between the delivery form to the centres, the randomisation list, and the number of kits returned will be made. A document tracking the return of the kits must be written.

Destruction

The destruction of the kits is under the responsability of the promoter. The presence of a pharmacist from another centre as a witness, as well as the authorization to be destroyed from the Regional Health Agency are required.

VI. - Measured variables and measurement methods:

The self-assessment of pain intensity by a numerical rating scale (NRS). The participant assigns a number between 0 and 10 to the intensity of pain he feels when the investigator asks him the question. 0 is the fact that he has no pain at all, 10 is the worst pain intensity imaginable. It is a validated and reproducible scale in emergency medicine.

Non-invasive blood pressure: Measured by a pressure cuff connected to an automated device (BP measurement every 10 minutes).

Heart rate and respiratory rate: Continuously monitored and collected every 10 minutes by the investigator on the observation logbook.

Oxygen saturation: Measured continuously by an automated device and collected every 10 minutes by the investigator on the observation logbook.

Sedation scale: score from 0 to 3 to rate the depth of a sedation. A score of 0 corresponds to a perfectly awake subject while a score of 3 corresponds to a subject in a state of coma, impossible to wake up whatever stimuli are used. (See Annex 3).

Subjective discomfort parameters (nausea, vomiting, bad taste, mucosal irritation, epistaxis...) Continuously monitored and collected by the investigator on the observation logbook.

Evaluation of the patient's pain management. The participant assigns a number between 0 and 10 for the assessment of pain management after 30 minutes. The number 0 corresponds to "not at all satisfied", 10 corresponds to optimal satisfaction.

VII - Data collection and management: observation booklet - quality control

The data are collected by the clinical investigators on a paper document (see Appendix 9) and transcribed by a Clinical Research Associate (CRA) into a specific software on a computer dedicated to the study.

To improve the quality of the collection of the data, the study team will be composed of several people (a clinical investigator, a medical student investigator's assistant and an emergency room nurse).

The investigator of each centre will enter the data from the paper documents into the software with the assistance (if necessary) of the CRA of the study. The same CRA will regularly check the quality of the data collection and correct any errors. He will also be responsible for checking the smooth running and safety of the kit circuit in the centres, mainly outside Grenoble University Hospital.

VIII. - Sample Collections:

No sample collection will be performed

IX. - Statistical analyses :

A- Places of analysis:

Grenoble University Hospital

B- Data analysis strategy:

a. Types of analyses :

This is a per Protocol analysis because ALGOFINE 2 is a non-Inferiority study.

b. Documentation of missing, unused or outliers data.

As the study requires an analysis of the per-protocol population, no replacement of missing data is considered for the analysis of the primary endpoint.

With regard to a possible analysis with the intention to treat, a multiple imputation method would be implemented. However, in order to avoid missing data, 3 people will be present during the inclusions (an investigator, a medical student, an emergency room nurse). This should help to reduce the risk. Nevertheless, we estimate that 10% of the files are likely to be incomplete.

c. Guarantee of data integrity

The data will be collected on specific software, available only to investigators (including a personalized password). The server automatically records the data in real time. These data will be available for consultation by the persons in charge of quality control. In addition, the paper data will be kept until the end of the study.

d- Statistical tests used for each analysis and their justification

The statistical analysis will be carried out after the implementation of the usual data management and database freezing procedures.

The descriptive analysis will cover all the subjects included. The qualitative parameters will be summarized in terms of numbers and absolute frequency. The quantitative parameters will be expressed by the mean and standard deviation or by the median and the 25th and 75th percentiles when normality has been rejected. The Shapiro-Wilks test will be used to demonstrate the normality of the parameters, and the Levene test to demonstrate the homogeneity of the variances. When the conditions for applying parametric tests are not met, non-parametric tests may be performed. All the tests used will be bilateral.

When analysing the main criterion, the statistical threshold = 2.5% will be used to consider a difference as statistically significant for each unilateral test. Concerning the parameters present in the observation book and whose analysis is not described, they will be used for descriptive purposes or in exploratory post-hoc analysis.

Analysis of the main objective:

The objective is to compare the efficacy of analgesia after 30 min with the reference treatment (Morphine IV) (non-inferiority).

The main criterion corresponds to the variation in the numerical pain scale (initial NRS - NRS at 30 min) between the reference treatment (Morphine IV) and the tested treatment (Sufentanil IN).

As this is a non-inferiority study, the analysis of the main objective will focus on the per-protocol population, i.e. all patients included in the emergency room without major violations of the protocol.

The non-inferiority of the treatment under study (Sufentanil IN) compared to the reference treatment (Morphine IV) in terms of variation in the numerical pain scale (initial NRS - NRS at 30 min) will be assessed using the Schuirmann test.[26]

The equivalence threshold corresponds to the minimum, clinically significant value of the evolution of the pain level; it has been defined a priori as a variation of 1.3 of the numerical scale (NRS)[24].

The statistical significance threshold will be $p<0.025$.

Analysis of secondary objectives:

Secondary objective 2:

The objective is to compare the efficacy of the analgesia performed by Sufentanil intra nasal compared to the reference treatment (Morphine IV) after 10 min and 20 min of treatment.

The criterion corresponds to the variation in the numerical pain scale (initial NRS - NRS at 10 min or 20 min) of each treatment (Sufentanil IN versus Morphine IV).

The analysis will be carried out on the per-protocol population and then on the Intent to Treat (ITT) population of patients hospitalized in emergency departments.

Evaluation of the effectiveness of pain analgesia at intermediate times will be performed between treatment groups. The comparative analysis of the variation in the numerical pain scale (initial NRS - final NRS (10 or 20 min)) will be carried out using the bilateral Student test. When the normality or homogeneity of variances is rejected, the Mann-Whitney non-parametric test will be implemented.

Secondary objective 3:

The objective is to evaluate the efficacy of analgesia performed by intra-nasal Sufentanil and IV Morphine after 10 min and 20 min of treatment.

The criterion corresponds to the measurement of the numerical scale (NRS) of pain at baseline and 10 and 20 min in each of the treatment groups (Sufentanil IN versus Morphine IV).

The analysis will be conducted on the per-protocol population and then on the Intended to Treat (ITT) population of patients hospitalized in emergency departments.

The evaluation of the effectiveness of pain analgesia at each intermediate time will be conducted according to the treatment group. The comparative analysis of the measurement of the numerical pain scale (10 or 20 min) will be performed using the Student's bilateral test for matched series in each group. When the normality or homogeneity of variances is rejected, the Wilcoxon non-parametric test will be implemented.

Secondary objective 4:

The objective is to compare the occurrence of adverse events between treatment groups (Sufentanil IN versus Morphine IV).

The criterion is the evaluation of the following adverse events in each of the treatment groups: Nausea, vomiting, epistaxis, nasal discomfort, deep sedation (sedation scale > 2), desaturation < 90%, blood pressure < 90 mm Hg, respiratory < 10 / min, use of Naloxone (antidote).

The analysis will be conducted on the per-protocol population and then on the Intended to Treat (ITT) population of patients hospitalized in emergency departments.

The comparative analysis of adverse events between treatment groups (Sufentanil IN versus Morphine IV) will be performed using the chi-2 test and by an accurate Fisher test when the expected value of a cell in the contingency table is less than 5.

Averse events will be expressed as percentage numbers and the 95% confidence interval will be associated. The significance threshold is set at $p<0.05$.

Secondary objective 5: Feasibility analysis of patients in pre-hospital care.

The objective is to evaluate the efficacy of the analgesia performed by Sufentanil intra nasal compared to the reference treatment (Morphine IV) after 10 min, 20 min and 30 min of treatment in the pre-hospital patient group.

The criterion corresponds to the variation in the numerical pain scale (initial NRS - NRS at 10 min, 20 min and 30 min) of each treatment (Sufentanil IN versus Morphine IV).

The analysis will be performed on the pre-hospital patient group of 30 subjects.

A descriptive evaluation of the effectiveness of pain analgesia at each time and the patient's satisfaction with its management will be performed between treatment groups. The comparative analysis of the variation in the numerical pain scale (initial NRS - final NRS (10, 20 or 30 min) will be carried out using a repeated measurement analysis of variance integrating, if necessary, the Greehouse-Geisser correction.

Secondary objective 6: patient satisfaction

The objective is to evaluate the satisfaction of the patient.

The criterion is the intensity of satisfaction assessed on a dedicated scale.

The comparative analysis of patient satisfaction will be performed using the bilateral Student test; when normality or homogeneity of variances is rejected, the Mann-Whitney non-parametric test will be implemented.

D- Persons responsible for the analyses:

-Writer of the statistical analysis plan:

Jean-Louis QUESADA, Biostatistician in the scientific department of the DRCI at the University Hospital of Grenoble.

-Persons responsible for carrying out the analysis:

The statistical analysis of the data will be carried out at Grenoble University Hospital in the scientific department of the DRCI by Mr. JL Quesada (Biostatistician) in collaboration with Dr. Marc Blancher.

E- Software

The statistical analysis will be performed using STATA® Software version 12 or higher (Stata Corporation 4905 Lakeway Drive College Station, TX 77845 USA).

X. - Calendar:

Study duration per patient: 4 hours

Total duration of the study 3 years

Anticipated start date of inclusions: September 1, 2013

Expected end date of inclusions: April 30, 2016

Expected end date of the study if different: August 31, 2016

XI. - Material and legal aspects:

Balance of benefits / risks of the study:

If the main hypothesis is verified, intra-nasal analgesia could be used to provide rapid relief to patients undergoing emergency management for acute trauma pain. This analgesia would be initiated more quickly for practical reasons and would expose patients to less risk of infection or pain associated with the placement of a venous catheter. Second, it would pave the way to pre-hospital intra-nasal analgesia for situations where it is unthinkable or even impossible to gain a venous access.

There is therefore an expected benefit for the patient.

However, our study does not pose any additional risks to the participants. Indeed, there are two types of risks: those related to the products used (Morphine and Sufentanil) and those related to the route of administration.

With regard to products:

In both arms of our study, participants receive opioids, the most adverse event effect of which is central respiratory depression. When bradypnea occurs with or without desaturation, naloxone, the antidotal treatment of opioids, can be administered. All participants will have a venous access and close monitoring.

These are two different products (Morphine and Sufentanil) which belong to the same morphine family. If the doses of analgesics are not perfectly known from a theoretical point of view, strong scientific arguments can be made to consider that Sufentanil is about 1000 times more potent than Morphine. The doses to be administered are therefore 1000 times lower.[26] In practice, 1 mg of Morphine is equivalent to 1 microgram of Sufentanil.

The kinetics of the two products are not exactly the same: the half-life of Morphine is slightly longer than that of Sufentanil, which tends to limit the risks of adverse events with the latter over time. On

the other hand, since the action time is shorter, 1 to 2 min for Sufentanil, adverse events can occur earlier with Sufentanil than with Morphine. However, this does not constitute an additional risk since patients are particularly monitored during this phase of the protocol. Finally, the route of administration of the products is different and published clinical and pharmacological studies show that for the same product, the intra-nasal dose should be 1.4 times that of the intravenous.

With regard to the route of administration:
In theory, there is less risk of infection through the IN route than through the IV route since it is a non-invasive route. There is therefore no risk of catheter-related infection. Similarly, this approach is less painful since there is no puncture procedure. However, studies using Fentanyl IN have reported cases of bad taste in the mouth and there are two cases reported in the literature of nasal mucosal ulceration. No specific data on Sufentail is available.

Conclusion on the benefit/risk balance:
Considering that there are no more side effects described to date with the use of the intra-nasal route; that Sufentanil and Morphine have the same risks of adverse reactions;
Considering also that the efficacy of analgesia obtained after intra-nasal spraying is identical (in the literature) to that obtained by IV route;
Considering further that the use of the Intra-nasal route would shorten the time required to administer analgesia to patients admitted to emergency departments (especially children);
Taking into account that there is a real need for the use of a non-invasive route of analgesia (especially in hostile environments);
It is lawful to consider that the benefit expected for this study is greater than the risks involved.

Ethical and regulatory provisions:
The research will be conducted in compliance with current French regulations, in particular the provisions relating to biomedical research of the Public Health Code, article L 1121-1and following, the Bioethics laws, the Data Protection Act, the Helsinki Declaration, as well as the Good Clinical Practices.
This protocol will be authorised by the promoter after a favourable opinion from the Comité de Protection des Personnes (C.P.P.) Sud-Est V, authorized by the Agence Nationale de Sécurité des Médicaments et des produits de santé (ANSM ex AFSSAPS). It is covered by civil liability insurance taken out by the promoter with the Société Hospitalière d'Assurances Mutuelles (SHAM- 18, rue Edouard Rochet - 69 372 LYON cedex08).

Subject Information - Written Informed Consent

In accordance with Good Clinical Practices and current legal provisions, any pre-selected patient will be informed in advance by the investigator of the objectives of the study, its methodology, duration, constraints and foreseeable risks, possible therapeutic alternatives, and the medical management procedures planned at the end of the research, including in the event that the study is stopped before its end. In particular, it will be made clear to the subject that he or she is entirely free to refuse to participate in the study or to withdraw his or her consent at any time without incurring any liability or prejudice as a result. A document summarizing the information provided by the investigator will be provided.

After ensuring that the information provided is well understood, the investigator will seek the subject's written consent to participate in the study. If he/she agrees, the volunteer will sign the consent form before the study is conducted.

Professional secrecy, confidentiality

The investigator is bound by professional secrecy. The data collected, including the results of the analyses, shall be made anonymous by any appropriate means. The sponsor and its representatives are subject to the same professional secrecy obligations as the investigator. This document and its annexes are given to the investigator on a confidential basis and should only be given or communicated to persons specifically involved in the trial with the agreement or at the request of the coordinator, the sponsor, and possibly a representative of the Health Authorities.

This study benefits from the declaration made by the University Hospital of Grenoble, of compliance with the MR-001 reference methodology of the CNIL (French Authority for data protection) for the processing of personal data implemented in the context of biomedical research.

In accordance with the law known as "Informatique et Libertés", patients will be informed that the data will be processed in a non-nominative and computerised way and that they will be able to access the data concerning them either with the investigator or through the intermediary of the physician of their choice. (Law of 6 January 1978 amended relating to data processing, files and freedoms).

Financing of the study

This study is funded by the French Society of Emergency Medicine and Nycomed Takeda Laboratories

Amendments to the Protocol

Any substantial modification of the protocol shall be the subject of an amendment submitted to the ethic committee, and to the competent authority for their opinion and authorization.

Anonymity of the subjects participating in the study

The observation booklet slips will only bear the patient's initials (first three letters of the surname and first two letters of the first name) and an anonymity number. Only this number will be computerized.

Quality assurance

The clinical part of the study will be carried out in accordance with Good Clinical Practices.

Data monitoring

A copy of the observation booklet (or CRF Case Report Form) prepared for the study must be sent to the proponent as soon as it is prepared. Concerning the electronic case report form, the access codes for all read-only data must be transmitted to the promoter

A data monitoring will be carried out regularly by a Clinical Research Associate. All consents will be verified, as well as inclusion and non-inclusion criteria, and adverse events. Data monitoring will cover 20% of the observation books selected at random.

A monitoring report will be prepared by the CRA and kept in the study file.

During site visits, the monitor will check the proper storage conditions for treatments.

XII- Adverse event(s)

A- Definitions of the terms

a. Adverse event (AE)

Any harmful manifestation occurring in a person who is engaged in biomedical research, whether or not related to research or experimental drug(s), medical devices, labile blood products, human body products used for therapeutic purposes, cell therapy products, cosmetics or tattoos on any person carrying out such research.

b. Serious Adverse Event (SAE):

A serious adverse event is an event whose evolution is fatal, or that endangers the life of the person who is the subject of the research, or that results in a significant or lasting disability or handicap, or that causes hospitalization or prolongation of hospitalization or that results in a congenital anomaly or malformation or any other event not meeting the qualifications listed above, but which may be considered as "potentially serious", in particular certain biological anomalies or medically relevant event as determined by the investigator, or an event requiring medical intervention to prevent progression to one of the precipitated states.

Any SAE listed in the protocol (RCP Sufentanil and Morphine (Chlorydrate)) is considered an expected SAE. Conversely, any SAE not mentioned is considered as an unexpected SAE.
In order to comply with the regulations in force concerning the reporting of serious adverse reactions to health authorities, the investigator undertakes to document the event, to respect the reporting deadlines and to provide all the information necessary for the analysis of this event.

Thus, for each adverse event, the investigator should
-notify as soon as possible all the SAE that occurred in the trial using the declaration form annexed to the electronic CRF and annexed to the protocol. -give an etiological diagnosis
-assess their severity
-assess their intensity
-establish a causal link between the experimental product and the SAE
-provide the promoter with relevant additional information on the SAE within 8 days of the declaration.
-monitor the patient who has presented a SAE until it is resolved, stabilized to a level acceptable to the investigator or returned to the previous state, even if the patient has been discharged from the trial.

Any ISAE must therefore be declared by the investigator to the structure in charge of the promoter's vigilance:
Regional Pharmacovigilance Centre,
To Dr Edith SCHIR or Dr Adeline Paris, Pavillon E, Grenoble University Hospital.
Tel: 04 76 76 51 45 Fax: 04 76 76 56 55
E-mail: ESchir@chu-grenoble.fr or vigilance-essaiscliniques@chu-grenoble.fr),
regardless of its causal relationship with the treatment(s) of the trial or research as soon as it becomes aware of it within the time limits required by the sponsor.

B- Specificities of the ALGOFINE 2 study

a. Non-serious adverse events

All non-serious adverse events and/or adverse analytical findings, defined in the protocol as relevant to the safety assessment of persons participating in the clinical trial, should be reported to the sponsor by the investigator as soon as possible to allow for continuous benefit/risk balance analysis and group comparison.
They are noted in the data collection book and the information will be made available to the pharmacovigilance department of the clinical trials.

Allergic reaction
Defective product
Epistaxis, rhinorrhea (discomfort related to the smell of the intra-nasal product) Nausea / Vomiting
Hot flashes
Hallucinations

b. Serious events to be reported immediately
PAS < 90 mm Hg
Heart rate < 50 / min
Sedation Score > 2
Respiratory rate < 10 / min
SpO2 < 90%
Anaphylactic shock
Septic shock
Infection of the IV injection site
Nasal ulceration

Independent Oversight Committee
As provided for in article L1123-7, paragraph 2 of the CSP, an independent monitoring committee for the clinical trial has been set up. It will be chaired by Dr D. Anglade (Department of Anesthesia - Resuscitation, Chairman of the Pain Control Committee) and composed of Pr J.L Cracowski (Clinical Investigation Centre) and Dr C. Villier (Pharmaco Vigilance). The members of this committee validated their participation by signing a confidentiality agreement and declaration of no conflict of interest. It will meet after 100 patients included and conduct a descriptive study to assess the safety of the study. It will also meet in the event of serious adverse events with an unusual frequency or at the request of the structure in charge of the vigilance of the trial.
The protocol will then only be continued after this committee has given its opinion. This committee may propose to the sponsor and the coordinating investigator that this research be stopped or that the protocol be modified if the safety of the subjects suitable for the study does not seem sufficient.

XIII - Early termination of research:

A- Criteria for stopping the study for a subject participating in the study
A subject who withdraws his or her consent to participate in the study. Allergy to the product used.
Any change in the patient's health condition that results in the patient no longer meeting the inclusion criteria

Occurrence of unexpected Serious Adverse Events

B- Premature cessation of treatment by the subject

Criteria and procedures for stopping treatment: The subject no longer wishes to take the treatment.

Modality of patient follow-up after stopping treatment: The subject's pain will be relieved by following the usual protocol of the emergency department of the hospital centre where the investigation is being conducted. The subject's medical follow-up remains the same if he or she stops taking the treatment planned in the study.

Modality of replacement of these persons A Substantial modification may be submitted to increase the number of inclusions if statistically the number of subjects with usable data is not sufficient.

C- Stopping the study by the proponent:

The sponsor may stop the study at any time for the following reasons:

Investigator's inability to include patients according to schedule

No signed consent

Major violations of the protocol

Incomplete or incorrect data

D- Stopping the study by the investigator

In the event of an adverse event considered by the investigator to be severe and likely to affect the health of patients, the investigator may stop the study in agreement with the sponsor.

XIV - Archiving:

All documents and data relating to the study will be archived for a period of 15 years by the investigator under the responsibility of the sponsor. The list of essential documents that make up the permanent research file is detailed in paragraph 8 of the Good Clinical Practices.

XV - Publication

This protocol will be the subject of a declaration on the website www.clinicaltrials.gov.

All data collected during this study are the property of the study sponsor and may not be disclosed to any third party under any circumstances without the written consent of the sponsor and the investigator. Any publication or communication (oral or written) will be decided by mutual

agreement between the investigators and will respect the international recommendations: "Uniforms Requirements for Manuscripts Submitted to Biomedical Journals". (http://www.icmje.org)

In all publications related to the study, the sponsor will appear in the acknowledgements.

XV - DATE AND SIGNATURES:

This protocol was read and approved on the date noted in the header

Investigator Coordinator

Dr Marc BLANCHER
SAMU / SMUR service

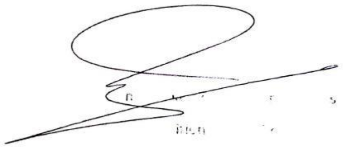

For the promoter

Mrs Jacqueline HUBERT
Managing Director
Grenoble University Hospital

XVI - Bibliographical references:

1. Trinh-Duc. 2007 update of the 2nd Emergency Medicine Consensus Conference: Drug treatment of adult pain in emergency settings. *Pain-Evaluation-Diagnosis-Treatment* **9**, 248-278 (2008).
2. Vivien, B., *et al.* [Sedation and analgesia in the emergency context]. *Ann Fr Anesth Reanim* **29**, 934-949 (2010).
3. Vora, K.S., Shah, V.R., Patel, B., Parikh, G.P. & Butala, B.P. Postoperative analgesia with epidural opioids after cesarean section: Comparison of Sufentanil, Morphine and Sufentanil-Morphine combination. *J Anaesthesiol Clin Pharmacol* **28**, 491-495 (2012).

4 Helmers, J.H., Noorduin, H., Van Peer, A., Van Leeuwen, L. & Zuurmond, W.W. Comparison of intravenous and intranasal Sufentanil absorption and sedation. *Can J Anaesth* **36**, 494-497 (1989).

5. Steenblik, J., *et al.* Intranasal Sufentanil for the treatment of acute pain in a winter resort clinic. *Am J Emerg Med* **30**, 1817-1821 (2012).
6. Stephen, R., Lingenfelter, E., Broadwater-Hollifield, C. & Madsen, T. Intranasal Sufentanil provides adequate analgesia for emergency department patients with extremity injuries. *J Opioid Manag* **8**, 237-241 (2012).
7. Mercadante, S., *et al.* A comparison of intranasal Fentanyl spray with oral transmucosal Fentanyl citrate for the treatment of breakthrough cancer pain: an open-label, randomised, crossover trial. *Curr Med Res Opin* **25**, 2805-2815 (2009).
8. Striebel, H.W., Pommerening, J. & Rieger, A. Intranasal Fentanyl titration for postoperative pain management in an unselected population. *Anaesthesia* **48**, 753-757 (1993).
9. Striebel, H.W., Oelmann, T., Spies, C., Rieger, A. & Schwagmeier, R. Patient-controlled intranasal analgesia: a method for noninvasive postoperative pain management. *Anesth Analg* **83**, 548-551 (1996).
10. Toussaint, S., Maidl, J., Schwagmeier, R. & Striebel, H.W. Patient-controlled intranasal analgesia: effective alternative to intravenous PCA for postoperative pain relief. *Can J Anaesth* **47**, 299-302 (2000).
11. Christrup, L.L., Foster, D., Popper, L.D., Troen, T. & Upton, R. Pharmacokinetics, efficacy, and tolerability of Fentanyl following intranasal versus intravenous administration in adults undergoing third-molar extraction: a randomized, double-blind, double-dummy, two-way, crossover study. *Clin Ther* **30**, 469-481 (2008).
12. Finn, M. & Harris, D. Intranasal Fentanyl for analgesia in the paediatric emergency department. *Emerg Med J* **27**, 300-301 (2010).
13. Rickard, C., *et al.* A randomized controlled trial of intranasal Fentanyl vs intravenous Morphine for analgesia in the prehospital setting. *Am J Emerg Med* **25**, 911-917 (2007).

14. Holdgate, A., Cao, A. & Lo, K.M. The implementation of intranasal Fentanyl for children in a mixed adult and pediatric emergency department reduces time to analgesic administration. *Acad Emerg Med* **17**, 214-217 (2010).
15. Borland, M., Jacobs, I., King, B. & O'Brien, D. A randomized controlled trial comparing intranasal Fentanyl to intravenous Morphine for managing acute pain in children in the emergency department. *Ann Emerg Med* **49**, 335-340 (2007).
16. Illum, L. Is nose-to-brain transport of drugs in man a reality? *J Pharm Pharmacol* **56**, 3-17 (2004).
17. Pires, A., Fortuna, A., Alves, G. & Falcao, A. Intranasal drug delivery: how, why and what for? *J Pharm Pharm Sci* **12**, 288-311 (2009).
18. Dale, O., Hjortkjaer, R. & Kharasch, E.D. Nasal administration of opioids for pain management in adults. *Acta Anaesthesiol Scand* **46**, 759-770 (2002).
19. Mathieu, N., Cnudde, N., Engelman, E. & Barvais, L. Intranasal Sufentanil is effective for postoperative analgesia in adults. *Can J Anaesth* **53**, 60-66 (2006).
20. Henderson, J.M., Brodsky, D.A., Fisher, D.M., Brett, C.M. & Hertzka, R.E. Pre-induction of anesthesia in pediatric patients with nasally administered Sufentanil. *Anesthesiology* **68**, 671-675 (1988).
21. Borland, M.L., Clark, L.J. & Esson, A. Comparative review of the clinical use of intranasal Fentanyl versus Morphine in a paediatric emergency department. *Emerg Med Australas* **20**, 515-520 (2008).
22. Crellin, D., Ling, R.X. & Babl, F.E. Does the standard intravenous solution of Fentanyl (50 microg/ml) administered intranasally have analgesic efficacy? *Emerg Med Australas* **22**, 62-67 (2010).
23. Lundeberg, S. & Roelofse, J.A. Aspects of pharmacokinetics and pharmacodynamics of Sufentanil in pediatric practice. *Paediatr Anaesth* **21**, 274-279 (2011).
24. Fleischman, R.J., Frazer, D.G., Daya, M., Jui, J. & Newgard, C.D. Effectiveness and safety of Fentanyl compared with Morphine for out-of-hospital analgesia. *Prehosp Emerg Care* **14**, 167-175 (2010).
25. Borland, M., Milsom, S. & Esson, A. Equivalency of two concentrations of Fentanyl administered by the intranasal route for acute analgesia in children in a paediatric emergency department: a randomized controlled trial. *Emerg Med Australas* **23**, 202-208 (2011).
26. Schuirmann, D. A.. A comparison of the two one-sided tests procedure and the power approach for assessing the equivalence of average bioavailability. Pharmacometrics. 15, 657-680 (1987).

27. Birnbaum A., Esses D., Randomized Double-Blind Placebo-Controlled Trial of Two Intravenous Morphine Dosages (0.10 mg/kg and 0.15 mg/kg) in Emergency Department Patients With Moderate to Severe Acute Pain. 49:445-453 (2007).
