# Supplementary material for: Intranasal sufentanil versus intravenous morphine for acute severe trauma pain: A double-blind randomized non-inferiority study
Source: PLoS Med. 2019 Jul 16;16(7):e1002849. doi: 10.1371/journal.pmed.1002849 (PMC6634380; doi:10.1371/journal.pmed.1002849)

Etude ALGOFINE-2

ANALGESIE AU SUFENTANIL PAR VOIE INTRA NASALE
VERSUS MORPHINE IV AUX URGENCES

Etude randomisée, contrôlée en double aveugle, double placebo, multicentrique

Intranasal Sufentanil analgesia
versus Morphine intravenous in Emergency room

A multi-center, randomized, controlled, double-blind, double placebo trial

Plan d'analyse statistique de l'étude

(Statistical analysis plan of the study)

Investigateur coordonnateur : Dr Marc Blancher

Promoteur : CHU de Grenoble

Responsable scientifique : Dr Raphael Briot

Centre d'Investigation Clinique

Auteur du plan d'analyse statistique : Jean-Louis Quesada

1. ADMINISTRATIVE INFORMATION 4

1a – Title and trial registration - Descriptive 4
1b – Title and trial registration - Trial registration number 4
2 – SAP version 4
3 – Protocol version 4
4a – SAP revisions - History 4
4b – SAP revisions - Justification for each SAP revision 6
4c – SAP revisions - Timing of SAP revisions in relation to interim analyses 7
5 – Roles and Responsibility 8

2. INTRODUCTION 8

7 – Background and aim 8
8 – Objectives 9

3. STUDY METHODS 10

9 – Trial Design 10
10 – Randomization 10
11 – Sample size 10
12 – Framework 11
13a – Statistical interim analyses and stopping guidance - Information 11
13b – Statistical interim analyses and stopping guidance – planned adjustment 11
13c – Statistical interim analyses and stopping guidance – Details of guidelines 11
14 – Timing of outcome analysis 11
15 – Timing of outcome assessments 12

4. STATISTICAL PRINCIPLES 12

16 – Confidence intervals and P values - Level of statistical significance 12
17 – Confidence intervals and P values - Description 12
18 – Confidence intervals and P values – Confidence intervals to be reported 12
19a – Adherence and protocol deviations – Definition of adherence 12
19b – Adherence and protocol deviations – Description of adherence 13
19d – Adherence and protocol deviations – Description of protocol deviations to be summarized 13
20 – Analysis populations 13

5. TRIAL POPULATION 13

21 – Screening data 13
22 – Eligibility 13
23 – Recruitment 14
24a – Withdrawal / follow-up - Level 14
24b – Withdrawal / follow-up – Timing / Lost of follow-up data 15
24c – Withdrawal / follow-up – Reasons and details 15
25a – Baseline patient characteristics – List of baseline patient characteristics to be summarized 15
25b – Baseline patient characteristics – Details 15

6. ANALYSIS 16

26a – Outcome definitions – specifications and timing 16
26b – Outcome definitions – measurement and units 16
26c – Outcome definitions – transformation used 16
27a – Analysis methods - presentation 16
27b – Analysis methods – adjustment for covariates 18
27c – Analysis methods –assumptions to be checked 18
27d – Analysis methods – alternative methods 19
27e – Analysis methods – planned subgroup analyses 20
28 – Missing Data 20
29 – Additional analyses 20
30 – Harms 21

31 – Statistical software ... 21
32a – References – for non-standard statistical methods ... 21
32b – References to Data Management Plan ... 21
32c – References to the Trial Master File ... 21
32d – References to other standard ... 21

7. APPENDIX I ... 22

Template of table of descriptive analysis at the inclusion visit ... 22
Templates of table of the principal outcomescales analysis between the inclusion (T0) and the visit at T30 and between groups. ... 23
Templates of tables of scales analysis between the inclusion (T0) and the visit at T10 or T20 and between groups. ... 23
Templates of tables of scales analysis between the inclusion (T0) and the visit at T10 by groups. ... 23
Templates of tables of scales analysis between the inclusion (T0) and the visit at T20 by groups. ... 24
Template of table of adverse event analysis between groups. ... 25

8. APPENDIX II ... 26

Figure 1. Flow chart of patient enrollment ... 26

The analysis plan for this randomized trial was formatted to conform to the 2017 version of the recommendations "Guidelines for the content of statistical analysis plans in clinical trials".

1. Administrative Information

1a – Title and trial registration - Descriptive

Statistical analysis plan for the Algofine-2 study (« Intranasal Sufentanil analgesia versus Morphine intravenous in Emergency room »): A multi-center, randomized, controlled, double-blind, double placebo trial.

1b – Title and trial registration - Trial registration number

Trial Registration : EudraCT registration number : 2013-001665-16, Date 2013-08-01

2 – SAP version

Version 3.0 of the statistical analysis plan was carried out after the blind review of May 12, 2016 in the presence of Dr Marc Blanchet (Investigator, coordinator of the study), Dr Raphael Briot (Scientific Manager of the study), Cyrielle Clapé (CRA, Project Manager), Dr Damien Viglino (CCA) and Jean-Louis Quesada (Biostatistician), in accordance with the database freezing procedure.

This analysis plan was presented during the methodology EPP staff (Evaluation of Professional Practices) of the CIC of June 27, 2016. A sensibility analysis has been proposed, in accordance with the latest JAMA recommendations, to complete the analysis of the primary endpoint performed on the per protocol population by validating the results on the Intent to Treat (ITT) population.

3 – Protocol version

This document has been written based on information contained in the study protocol MS4 version 10, dated October 22nd, 2015.

4a – SAP revisions - History

Protocol version	Updated SAP Version number	Section number changed	Description of and reason for change	Date
7.0	1.0	-	Initial submission of the protocol to the Ethics Comity (French CPP) and French ANSM authorities.	31/07/2013

8.0 MS2	2.0	- Summary Section "main judgment criterion"	- Clarification of the main judgment criterion : Variation of the numerical scale of pain (EN at T30-EN at T0)	29/04/2014
		- Summary Section "target population"	- Clarification of the target population: distinction between the analysis population related to the main objective (2x110) and that linked to the secondary objective n°5 (30 complementary subjects).	
		- Summary Section "Data processing"	- Precision for the non-inferiority analysis: the threshold selected for this analysis will be 1.3.<br>- Precision for the statistical threshold of significance retained for the non-inferiority analysis (2.5%).<br>- General description of the descriptive and comparative analysis of secondary objectives.	
		- Chapter II Section "Objectives"	- Modifications and addition of objectives and secondary criteria.	
		- Chapter IV Section "sample size"	- Changes in sample size calculation: Change in the common standard deviation (2.1 to 2.8) related to the main criterion based on the publication *"Birnbaum A., Esses D., Randomized Double-Blind Placebo-Controlled Trial of Two Intravenous Morphine Dosages (0.10 mg/kg and 0.15 mg/kg) in Emergency Department Patients With Moderate to Severe Acute Pain. 49:445-453 (2007)"* and the reduction of the power of the analysis to 80%.	
		- Chapter IX Section "Replacement of missing data"	- Clarification on the replacement of missing data: For per-protocol population analysis, no replacement of missing data is considered for the analysis of the primary endpoint.<br>With regard to a possible intention-to-treat analysis, a multiple imputation method would be implemented.	
		- Chapter IX Section "Statistical tests"	- Clarification of Statistical Analysis: For each objective of the study, details of the statistical tests used and their justifications.<br>- Precision: A detailed analysis plan covering all objectives may be completed before the study database is frozen, in conjunction with the scientific steering committee of the study.	

-	3.0	"Superiority analysis"	- Clarification of Statistical Analysis: The evaluation of the superiority of intra-nasal Sufentanil compared to the reference treatment will be used if non-inferiority is demonstrated.	12/05/2016
-	3.1	"Primary endpoint analysis"	- Clarification of Statistical Analysis: A sensibility analysis has been proposed by the methodology EPP staff to complete the analysis of the primary endpoint performed on the per protocol population by validating the results on the Intent to Treat (ITT) population.	27/06/2016
-	3.2	-	SAP reviewed to take account of the new Guidelines (JAMA, 2017).<br>- Clarification of Statistical Analysis: The reviewer of the manuscript proposed to complete the analysis with a linear regression that adjusts for baseline pain score and site	13/05/2019

4b – SAP revisions - Justification for each SAP revision

The statistical analysis plan (SAP) of the clinical protocol has been updated April 29, 2014, to adapt it to:

- the clarification of the main judgment criterion : Variation of the numerical scale of pain (EN at T30 – EN at T0),
- precision for the non-inferiority analysis : the threshold selected for this analysis will be 1.3.
- precision for the statistical threshold of significance retained for the non-inferiority analysis (2.5%).
- general description of the descriptive and comparative analysis of secondary objectives.
- the clarification of the target population : distinction between the analysis population related to the main objective (2x110) and that linked to the secondary objective n°5 (30 complementary subjects).
- the modifications and addition of objectives and secondary criteria.
- the modification of the sample size according to the change in the common standard deviation (2.1 to 2.8) related to the main criterion based on the publication "*Birnbaum A., Esses D., Randomized Double-Blind Placebo-Controlled Trial of Two Intravenous Morphine Dosages (0.10 mg/kg and 0.15 mg/kg) in Emergency Department Patients With Moderate to Severe Acute Pain. 49:445-453 (2007)*" and the reduction of the power of the analysis to 80%.

- Clarification on the replacement of missing data: For per-protocol population analysis, no replacement of missing data is considered for the analysis of the primary endpoint.
- With regard to a possible intention-to-treat analysis, a multiple imputation method would be implemented.
- Clarification of Statistical Analysis: For each objective of the study, details of the statistical tests used and their justifications.
- Precision: A detailed analysis plan covering all objectives may be completed before the study database is frozen, in conjunction with the scientific steering committee of the study.

The Statistical Analysis Plan (SAP) of the clinical protocol was updated after the blind review on May 12, 2016, in accordance with the database freezing procedure, to incorporate a superiority analysis on the Intent to Treat (ITT) population in case of non-inferiority demonstration.

The Statistical Analysis Plan (SAP) of the clinical protocol was updated after the methodology EPP staff (Evaluation of Professional Practices) of the CIC of June 27, 2016. A sensibility analysis has been proposed, in accordance with the latest JAMA recommendations, to complete the analysis of the primary endpoint performed on the per protocol population by validating the results on the Intent to Treat (ITT) population.

The statistical analysis plan for this randomized trial was formatted May 13, 2019 to conform to the 2017 version of the recommendations “Guidelines for the content of statistical analysis plans in clinical trials” (JAMA, 2017).

4c – SAP revisions - Timing of SAP revisions in relation to interim analyses

Not applicable.

5 – Roles and Responsibility

Name	Affiliations	Roles of SAP contributors
Jean-Louis Quesada	Biostatistician methodologist<br>Plurithematic Clinical Investigation Center<br>(INSERM CIC P 1406)<br>CHU de Grenoble - Hôpital A.Michallon<br>BP 217<br>38043 Grenoble Cedex 09	Write the SAP for the Initial submission, and the updated of the protocol.
Staff EPP	Plurithematic Clinical Investigation Center<br>(INSERM CIC P 1406)<br>CHU de Grenoble - Hôpital A.Michallon<br>BP 217<br>38043 Grenoble Cedex 09	Reviewed and proposed complementary analysis to the SAP.
Dr Marc Blanchet	Principal Investigator coordinator	Approved the SAP.
Dr Raphael Briot	Manager Scientist	Approved the SAP.
Dr Damien Viglino	Methodologist	Approved the SAP.

2. Introduction

7 – Background and aim

Analgesia is one of the main objectives of the management of patients presenting in an emergency department. The traumatology is more particularly associated with acute pain episodes, this is the case at the time of the trauma but also when the traumatized area is solicited (removal of clothing, clinical or radiological examination). The Grenoble basin and the Alpine arc have the dual feature of being both densely populated in terms of population and geographically located very close to mountain areas. As a result, in addition to "classical" sports and domestic traumatology, mountain sports, and in particular winter sports, are added. If the pain symptom is particularly associated with trauma, it is also very often underestimated, which can have deleterious consequences on the clinical state of the patients.

Reducing these post-traumatic acute pain episodes is one of the public health goals that has been clearly defined by the Ministry of Health.

Other products derived from the Morphine are usable and have a theoretical interest because they act faster. This is the case of Sufentanil, which has a shorter reaction time (60 to 120 seconds) than Morphine[3].
In addition, Sufentanil has the advantage of a wider therapeutic margin than Morphine or Fentanyl. In practice, the studies carried out reveal little undesirable effects when using Sufentanil, often at doses higher than the equi-analgesia Morphine[4, 5, 6].

Studies published so far tend to show the efficacy and safety of Sufentanil used intranasally in the management of pain in emergencies. However, there are no randomized, controlled, blinded, emergency department studies in an adult population.
(Nota: from the Paragraph "justification of the study" of the protocol)

8 – Objectives

The main objective of the study is to compare the efficacy of analgesia performed by intra nasal Sufentanil compared to the reference treatment (IV Morphine).

The aim of the secondary objectives of the trial is to :

- Compare the efficacy of analgesia performed by intranasal Sufentanil compared to the reference treatment (IV Morphine) after 10 min and 20 min of treatment.
- Evaluate the efficacy of analgesia performed by intranasal Sufentanil and Morphine IV, after 10 min and 20 min of treatment.
- Compare the occurrence of adverse effects between treatment groups (Sufentanil IN versus Morphine IV).
- Evaluate the efficacy of analgesia performed by intranasal Sufentanil compared to the reference treatment (IV Morphine) after 10 min, 20 min and 30 min of treatment in the group of pre-hospital patients.
- Evaluate patient satisfaction

3. Study Methods

9 – Trial Design

This study is a multicentre prospective, randomized, double-blind, controlled, double placebo trial.

10 – Randomization

Randomization 1:1 in parallel groups was pre-performed in blocks of random sizes and stratified by centre. The randomization list was handed over to the central pharmacy in charge of preparing packs. The randomization list was generated usung the Nquery Advisor software version 7.0 (Statistical Solutions Ltd, Boston MA, USA).

11 – Sample size

Version 1.0 of July 31 2013 J-L Quesada (Protocol version 7.0 – 31/07/2013):

The number of patients to be included in the study was calculated for a non-inferiority study according to the following assumptions: The mean value of EN at 30 minutes in patients with pain ≥ 6, relieved by Morphine is 3.7 (standard deviation of 2.2). Given an alpha risk of 2.5% and a non-inferiority threshold of 1.3 (EN standard equivalence threshold usually accepted) [24]. The number of subjects required, calculated using the Schuirmann[25] method, is 99 in each group to be able to conclude that the evaluated treatment is non-inferior compared to the reference treatment on EN at 30 minutes with a power of 97%.

Taking into account the fact that this study is multi-centric and considering that 10% of the randomized patients are likely not to fulfill the criteria of the per protocol analysis, the total number of the study is therefore 220 patients (110 in each case group).

Version 2.0 of April 29, 2014 J-L Quesada (Protocol version 8.0 – 29/04/2014):

This study will involve a sample size of 198 patients (99 patients per group). In order to guarantee this number of analyzable patients, we envisage a complementary recruitment of 10 patients per group. The calculation of the number of patients required corresponds to a non-inferiority study design.

The null hypothesis corresponds to a difference in efficacy between treatment groups above the equivalence threshold; The alternative hypothesis allows us to conclude that the treatments are equivalent;

The primary endpoint is the difference, before / after, of the mean pain score assessed by a numerical scale at inclusion and 30 minutes after administration.
The number of subjects required was assessed by the Schuirmann method[26], taking into account a minimal, clinically significant value of pain level evolution defined a priori as a modification of 1.3 of the numerical scale (EN) [24], an alpha risk of 2.5% and a standard deviation of the response of 2.8[27].
A total of 99 subjects per group will thus be necessary to reject the null hypothesis of a difference in efficacy and to conclude that the evaluated treatment is not inferior compared to the reference treatment, in terms of the evolution of EN in the first 30 minutes, with a power of 80%.
This calculation was performed using the NQuery Advisor software version 7.0 (Statistical Solutions Ltd., Boston MA, USA).

Post-hoc analysis of the power of the study :
The recruitment was lower than expected over the planned duration of the study and the power remained above 80% with an effective standard deviation in reduction in pain by 2.1 in post-hoc analysis.

12 – Framework
The adopted test framework is based on non-inferiority hypothesis.

13a – Statistical interim analyses and stopping guidance - Information
No statistical interim was performed.

13b – Statistical interim analyses and stopping guidance – planned adjustment
Not applicable.

13c – Statistical interim analyses and stopping guidance – Details of guidelines
Not applicable.

14 – Timing of outcome analysis

The statistical plan analysis (SAP) for the final analysis of the trial was developed prior to database lock. The SAP will be reviewed and approved by an independent methodological comity and the principal investigator.

15 – Timing of outcome assessments

The parameters and scale were evaluated at different visits in accordance with the following table.

	Inclusion Randomisation T0	T10	T20	T30	T40	T50	
- Inclusion / exclusion criteria check	☑						
- Patient consent	☑						
- Demographic parameters.	☑						
- numerical scale of self-evaluation of pain (EN)	☑	☑	☑	☑	☑	☑	
- Non-invasive blood pressure	☑	☑	☑	☑	☑	☑	
- Heart rate and respiratory rate	☑	☑	☑	☑	☑	☑	
- Sedation score	☑	☑	☑	☑	☑	☑	
- Oxygen saturation and flow	☑	☑	☑	☑			
- The sedation scale	☑	☑	☑	☑			
- Injected and sprayed volumes	☑	☑	☑				
- Co-analgesia							☑
- Adverse Events							☑

4. *Statistical Principles*

16 – Confidence intervals and P values - Level of statistical significance

The threshold p $\leq$0.025 will be taken into account to define the significance of the statistical tests.

17 – Confidence intervals and P values - Description

No adjustment for multiplicity will be taken into account.

18 – Confidence intervals and P values – Confidence intervals to be reported

The exact method will be taken into account to calculate the confidence interval.

19a – Adherence and protocol deviations – Definition of adherence

Adherence to the protocol will be defined as compliance with the criteria for inclusions and exclusions of the protocol and adherence to treatment.
Premature discontinuation of study treatment will be considered as a non-adherence to the protocol.

19b – Adherence and protocol deviations – Description of adherence

Adherence to the protocol will be listed in the CONSORT flow diagram.

19d – Adherence and protocol deviations – Description of protocol deviations to be summarized

Premature discontinuation of study treatment will be recorded as a protocol deviation and listed in the CONSORT flow diagram.

20 – Analysis populations

The PP population will consist of all randomized patients without any major deviation from the protocol. The following deviations from protocol will lead to exclusion from the PP population:

- Subject withdrawing consent to participate in the study.
- Non-adherence to the protocol (Overdosing, underdosing, discontinued intervention).
- No respect of inclusion criteria.

The ITT population will consist of all patients who have been randomized. Patients will be analyzed in the study arm assigned.

5. *Trial Population*

21 – Screening data

Numbers of patients assessed for eligibility with acute traumatic pain will be presented in the CONSORT flow Diagram.

22 – Eligibility

All adults presenting to the emergency department with pain due to recent trauma and self-rated intensity ≥ 6 on the numeric pain scale.
This pain can be either permanent or triggered or aggravated paroxysmally by movements or mobilizations.
Proposed subjects for the study will meet each of the following criteria:

- Age between 18 and 75 years.
- Person affiliated with social security or beneficiary of such a scheme.
- Informed and written consent signed by the patient or their legal representative.

Subjects that meet at least one of the following criteria cannot be included:

- Age under 18 years or upper 75 years.
- Severe traumatized patient of grade A or B (cf Annex 6 classification TREUNAU).
- Pain of medical origin (eg chest pain, abdominal pain, headache ...).
- History of chronic respiratory, renal or hepatic insufficiency.
- Substance addiction.
- Pregnant woman / breastfeeding.
- History of medico-surgical sinus.
- SpO2 <90%.
- Systolic blood pressure <90 mmHg.
- Opioid Allergy.
- Patient with head trauma and neurological involvement (GCS score <14).
- Patient with facial massive trauma.
- Patient unable to understand or score pain EN.
- Patient who received an analgesic-type analgesic (level 3) within 6 hours before taking charge.
- ALR indication.
- Obstruction of nasal passages treated with nasal decongestants (particularly oxymetazoline) within 20 minutes of admission.
- Protected persons referred to in Articles L1121-5 to L1121-8 of the CSP.
- Uncontrolled epilepsy.
- Intracranial hypertension.
- Weight upper 100kg.

23 – Recruitment

Numbers of patients and reasons for withdrawal and/or exclusion from analysis will be presented in the CONSORT flow diagram.

24a – Withdrawal / follow-up - Level

Timing of withdrawals and patients lost to follow up will be presented.

24b – Withdrawal / follow-up – Timing / Lost of follow-up data

Timing of withdrawals and patients lost to follow up will be presented in CONSORT diagram format, with numbers and reasons for withdrawal and/or exclusion from analysis.

24c – Withdrawal / follow-up – Reasons and details

The reasons and details of the withdrawal / follow-up data presentation will be listed by categories (inadequate concentration in packaging kits, not meeting inclusion criteria, overdosing, underdosing, discontinued intervention).

25a – Baseline patient characteristics – List of baseline patient characteristics to be summarized

Patients will be describe at inclusion by age, gender, weight, trauma area (Head, shoulder, Arm/ elbow, Wrist / hand, Thorax wall, Rachis, Pelvis / hip, Leg / knee, Ankle or foot) vital parameters (Heart Rate, Respiratory Rate, Mean Arterial Pressure, numerical Rating Scale for pain), Co-analgesia (paracetamol, Codeine, Ketoprofen).

25b – Baseline patient characteristics – Details

Baseline and demographic characteristics will be summarized for PP population. Descriptive summary statistics will be used for reporting continuous (arithmetic median and Inter Quartile Rate) and categorical (numbers and percentages) variables.

6. *Analysis*

26a – Outcome definitions – specifications and timing

See section 15 « Timing of outcome assessments”.

26b – Outcome definitions – measurement and units

Not applicable

26c – Outcome definitions – transformation used

Not envisaged.

27a – Analysis methods - presentation

- Principal objective analysis :

The main objective is to compare the efficacy of analgesia after 30 min performed by intranasal Sufentanil compared to the reference treatment (Intravenous Morphine).

Outcome: The analysis criterion corresponds to the variation of the numerical scale of the pain (EN at T30 – EN at T0) between the reference treatment (Morphine IV) and the tested treatment (Sufentanil IN).

Population of analysis: The analysis of the main objective will focus on the per-protocol (PP) population, that is to say on all patients included hospitalized in emergencies without major violation to the protocol integrating in particular patients who received the treatment dose provided for in protocol.

Analysis: The non-inferiority of the study treatment compared to the reference treatment in terms of variation of the numerical scale of pain (EN at T30 – EN at T0) will be evaluated using a one sided mean-equivalence t test on the per protocol population. We used the TOSTT procedure (*Dinno A. 2015. tostt: mean-equivalence t tests, Stata software package*) that tests the equivalence of means in a symmetric equivalence interval using the two-tailed unilateral test approach (Schuirmann 1987)[26].

The equivalence threshold corresponds to the minimal, clinically significant, change in the level of pain; It has been defined a priori as a modification of 1.3 of the numerical scale (EN)[24].

The statistical threshold of significance will be p <0.025.

- Secondary objectives analysis :

- The objective n°2 is to compare the efficacy of analgesia performed by intranasal Sufentanil compared to the reference treatment (IV Morphine) after 10 min and 20 min of treatment.

Outcome: The analysis criterion corresponds to the variation of the numerical scale of the pain (EN at T10 or T20 – EN at T0) of each treatment (Sufentanil IN versus Morphine IV).

Population of analysis: The analysis will be carried out on the per-protocol (PP) population and then on the intention-to-treat (ITT) population of patients hospitalized in the emergency department.

Analysis: The evaluation of the efficacy of analgesia of pain at intermediate times will be carried out between treatment groups.

The comparative analysis of the variation of the numerical scale of the pain (EN at T10 or T20 – EN at T0) will be carried out using the two group Student t test (a two-sided test). The statistical threshold of significance will be $p < 0.05$.

- The objective n°3 is to evaluate the efficacy of analgesia performed by intranasal Sufentanil and Morphine IV, after 10 min and 20 min of treatment.

Outcome: The analysis criterion corresponds to the measurement of the numerical scale (EN) of pain at the initial and final times (EN evaluated at 10 and 20 min) in each of the treatment groups (Sufentanil IN versus Morphine IV).

Population of analysis: The analysis will be carried out on the per-protocol (PP) population and then on the intention-to-treat (ITT) population of patients hospitalized in the emergency department.

Analysis: The evaluation of the effectiveness of analgesia of pain at each intermediate time will be performed according to the treatment group.

The comparative analysis of the measurement of the numerical scale of the pain (10 or 20 min) will be carried out using the Student's test for paired series in each group (a two-sided test). The statistical threshold of significance will be $p < 0.05$.

- The objective n°4 is to compare the occurrence of adverse effects between treatment groups (Sufentanil IN versus Morphine IV).

Outcome: The analysis criterion corresponds to the evaluation of the following adverse effects in each of the treatment groups: Nausea, vomiting, epistaxis, discomfort in the nasal

passages, deep sedation (sedation scale> 2), desaturation <90%, pressure arterial <90 mmHg, respiratory rate <10 / min, use of Naloxone (antidote).

Population of analysis: The analysis will be carried out on the per-protocol (PP) population and then on the intention-to-treat (ITT) population of patients hospitalized in the emergency department.

Analysis: The comparative analysis of adverse effects between treatment groups (Sufentanil IN versus Morphine IV) will be performed using the chi-2 test and by an exact Fisher test when the expected value of a contingency table cell will be less than 5.

The statistical threshold of significance will be $p < 0.05$.

- The objective n°5 is to evaluate the feasibility in pre-hospitalized patients.

The objective is to evaluate efficacy of analgesia performed by intranasal Sufentanil compared to the reference treatment (IV Morphine) after 10 min, 20 min and 30 min of treatment in the group of pre-hospital patients. The objective is to evaluate patient satisfaction.

Outcome: The analysis criterion corresponds to the variation of the numerical scale of the pain (EN at 10 min, 20 min and 30 min – EN at T0) of each treatment (Sufentanil IN versus Morphine IV).

Population of analysis: The analysis will be performed on the pre-hospital patient group of 30 subjects.

Planned analysis: A descriptive evaluation of the effectiveness of analgesia of pain at each time and the satisfaction of the patient with regard to its management will be carried out between treatment groups. The comparative analysis of the variation of the numerical scale of the pain (EN at 10 min, 20 min and 30 min – EN at T0) will be carried out using a repeated measures analysis of variance integrating if necessary the correction from Greehouse-Geisser;

The recruitment of patients in the pre-hospital group is not sufficient to achieve this goal.

<u>27b – Analysis methods – adjustment for covariates</u>

- <u>Principal outcome analyzes (secondary objectives) :</u>

The reviewer of the manuscript proposed to complete the analysis with a linear regression that adjusts for baseline pain score and site (as a stratification variable) to give a more precise treatment effect estimation.

<u>27c – Analysis methods –assumptions to be checked</u>

The parameters normality will be checked using Shapiro Wilks test.

The variances homogeneity will be checked using test on the equality of standard deviations.

27d – Analysis methods – alternative methods

- Principal outcome analyzes :

The reviewer of the manuscript proposed to complete the analysis with a linear regression that adjusts for baseline pain score and site (as a stratification variable) to give a more precise treatment effect estimation.

- Principal outcome analyzes (complementary analysis) :

The superiority of intra-nasal Sufentanil compared to the reference treatment will be evaluated using a two sample Student t test after verification of the application conditions. However, in the case where the application conditions were not respected, a non-parametric Mann-Whitney test, considered robust, was considered as a possible alternative.

- Secondary outcome analyzes (secondary objectives) :

- The objective n°2 is to compare the efficacy of analgesia performed by intranasal Sufentanil compared to the reference treatment (IV Morphine) after 10 min and 20 min of treatment.

The efficacy of intra-nasal Sufentanil compared to the reference treatment will be evaluated using a non-parametric Mann-Whitney test, considered robust, when the application conditions of the two group Student t test will not be respected.

- The objective n°3 is to evaluate the efficacy of analgesia performed by intranasal Sufentanil and Morphine IV, after 10 min and 20 min of treatment.

The comparative analysis of the measurement of the numerical scale of the pain (10 or 20 min) compared to baseline will be performed using a non-parametric Wilcoxon test when the application conditions of the Student's test for paired series will not be respected.

- The objective n°4 is to compare the occurrence of adverse effects between treatment groups (Sufentanil IN versus Morphine IV).

The comparative analysis of the adverse effects between treatment groups (Sufentanil IN versus Morphine IV) will be performed using an exact Fisher test when the expected value of a contingency table cell will be less than 5.

- The objective n°5 is to evaluate the feasibility in pre-hospitalized patients.

The comparative analysis of patient satisfaction will be performed using a non-parametric Mann-Whitney test when the normality or homogeneity of the variances is rejected.

27e – Analysis methods – planned subgroup analyses

No subgroup analysis is considered for the randomized trial analysis.

28 – Missing Data

- Recoding Primary criteria:

Implementation of the modified ITT analysis required the replacement of missing data; this was performed using the multiple imputation method. Ten imputations of the missing data were taken into account in the multiple imputation process.

- Recoding Secondary and exploratory criteria:

No data replacement was considered.

29 – Additional analyses

- Principal outcome analyzes (sensibility analysis) :

Sensitivity analysis of the primary objective: The evaluation of the non-inferiority of intra-nasal Sufentanil compared to the reference treatment conducted on the per-protocol population (PP) will be confirmed on the intention-to-treat population (ITT) in sensitivity analysis to comply recommendations of the guidelines (JAMA, 2012). This will be evaluated using a Schuirmann test on the imputed data. This analysis will retain the 2.5% as the statistical threshold.

In practice for a more robust result, both analyzes (PP and ITT) must be performed and give similar results.

- Principal outcome analyzes (complementary analysis) :

The evaluation of the superiority of intra-nasal Sufentanil compared to the reference treatment is used in hierarchical analysis. This will be evaluated on the intention to treat analysis using a two sample Student t test after verification of the application conditions.

Although unilateral testing appears to be most consistent with therapeutic hypotheses, we used a two-sided test. By decreasing the risk of alpha error on the conclusion of superiority, a two-sided test is more conservative.

This analysis will retain the 5% as a statistical threshold. This analysis will not require adjustment of the statistical threshold.

30 – Harms

The safety data will be summarized by the frequency of events.

31 – Statistical software

Statistical analysis will be performed using STATA® Software Version 14.2 or higher (Stata Corporation 4905 Lakeway Drive College Station, TX 77845 USA).

32a – References – for non-standard statistical methods

Not applicable

32b – References to Data Management Plan

The data consistency check was performed during the data entry on Redcap and the blind review preparation.

32c – References to the Trial Master File

The Clinical Research Department (DRCI) of the University Hospital Center of Grenoble Alps (CHUGA) was in charge of maintaining and storing documents related to the "Algofine-2" clinical trial.

The Trial Master File is available from the DRCI under the reference 38RC13.558 ; This information is archived on the DRCI server.

32d – References to other standard

RCDMS.MOP.009 – GEL D'UNE BASE DE DONNEES.

RCDMS.FOR.012 – CERTIFICAT DE GEL DE BASE DE DONNEES.

Gamble C, Krishan A, Stocken D, Lewis S, Juszczak E, Doré C, Williamson PR, Altman DG, Montgomery A, Lim P, Berlin J, Senn S, Day S, Barbachano Y, Loder E. Guidelines for the Content of Statistical Analysis Plans in Clinical Trials. JAMA. 2017; 318(23):2337-2343.

7. Appendix I

Template of table of descriptive analysis at the inclusion visit

Table 1 – Baseline characteristics of participants

		Groupe 1<br>n=XXX	Groupe 2<br>n=XXX
Age	median [IQR]		
Gender (Men)	Frequency(number)		
Weight	median [IQR]		
**Trauma area**			
Head	Frequency(number)		
Shoulder	Frequency(number)		
Arm / elbow	Frequency(number)		
Wrist or hand	Frequency(number)		
Thorax wall	Frequency(number)		
Rachis	Frequency(number)		
Pelvis / hip	Frequency(number)		
Leg / knee	Frequency(number)		
Ankle or foot	Frequency(number)		
**Vital Parameters at inclusion**			
HR(per min)	median [IQR]		
RR (per min)	median [IQR]		
SpO_2 (%)	median [IQR]		
MAP (mmHg)	median [IQR]		
NRS (/10)	median [IQR]		
**Co-analgesia**			
Paracetamol	Frequency(number)		
Codeine	Frequency(number)		
Ketoprofen	Frequency(number)		

Templates of table of the principal outcomescales analysis between the inclusion (T0) and the visit at T30 and between groups.

Table 2 – Criteria between T0 and T30

Scale EN	Group 1 (n=XXX)	Group 2 (n=XXX)	Delta G1-G2 mean ± se [IC95%] Effect Size [IC95%]	p value
Delta EN T30-T0	mean ± SD	mean ± SD	mean ± SD [CI], Cohen's d Effect Size [CI]	P value(5)

Results expressed as mean ± standard deviation [95% CI], Cohen's d Effect Size [95% CI]
(5) p based on an Student two sample T test.

Templates of tables of scales analysis between the inclusion (T0) and the visit at T10 or T20 and between groups.

Table 3 – Criteria between T0 and T10 or T20

Scale EN	Group 1 (n=XXX)	Group 2 (n=XXX)	Delta G1-G2 mean ± se [IC95%]	p value
Delta EN T10-T0	mean ± SD	mean ± SD	mean ± SD [CI], [IC95%]	P value(5)
Delta EN T20-T0	mean ± SD	mean ± SD	mean ± SD [CI], [IC95%]	P value(5)

Results expressed as mean ± standard deviation [95% CI]
(5) p based on an Student two sample T test.

Templates of tables of scales analysis between the inclusion (T0) and the visit at T10 by groups.

Table 4 – Criteria between T0 and T10

Scale EN	T0	T10	Delta T10-T0 Effect Size [IC95%]	p value
**Groupe 1** (n=XXX)	mean ± SD	mean ± SD	mean ± SD [CI], [IC95%]	P value(5)
**Groupe 2** (n=XXX)	mean ± SD	mean ± SD	mean ± SD [CI], [IC95%]	P value(5)

Results expressed as mean ± standard deviation [95% CI]
(5) p based on an Student paired sample T test.

Templates of tables of scales analysis between the inclusion (T0) and the visit at T20 by groups.

Table 5 – Criteria between T0 and T20

Scale EN	T0	T20	Delta T20-T0<br>Effect Size [IC95%]	p value
**Groupe 1**<br>(n=XXX)	mean ± SD	mean ± SD	mean ± SD [CI],<br>[IC95%]	P value(5)
**Groupe 2**<br>(n=XXX)	mean ± SD	mean ± SD	mean ± SD [CI],<br>[IC95%]	P value(5)

Results expressed as mean ± standard deviation [95% CI]
(5) p based on an Student paired sample T test.

Template of table of adverse event analysis between groups.

Table 6 –Adverse events observed

	Groupe 1 n=XXX	Groupe 2 n=XXX	p value
**Severe adverse events**	number (Frequency)	number (Frequency)	p value
Hypoxemia (SpO_2 <90%)	number (Frequency)	number (Frequency)	p value
Hypotension (SBP <90 mmHg)	number (Frequency)	number (Frequency)	p value
Bradypnea (RR <10/min)	number (Frequency)	number (Frequency)	p value
Anaphylactic shock	number (Frequency)	number (Frequency)	p value
Alteration of consciousness (Ramsay>2)	number (Frequency)	number (Frequency)	p value
Bradycardia (bpm<45/min)	number (Frequency)	number (Frequency)	p value
Naloxone use	number (Frequency)	number (Frequency)	p value
**Mild adverse events**	number (Frequency)	number (Frequency)	p value
Dizziness	number (Frequency)	number (Frequency)	p value
Hot flushes	number (Frequency)	number (Frequency)	p value
Nausea or vomiting	number (Frequency)	number (Frequency)	p value
Bad taste / smell	number (Frequency)	number (Frequency)	p value
Mild allergic reaction	number (Frequency)	number (Frequency)	p value
Epistaxis / Rhinorrhea	number (Frequency)	number (Frequency)	p value
Hallucinations	number (Frequency)	number (Frequency)	p value

Results expressed in number (Frequency),
Chi2, Fisher's exact test

8. Appendix II

Figure 1. Flow chart of patient enrollment

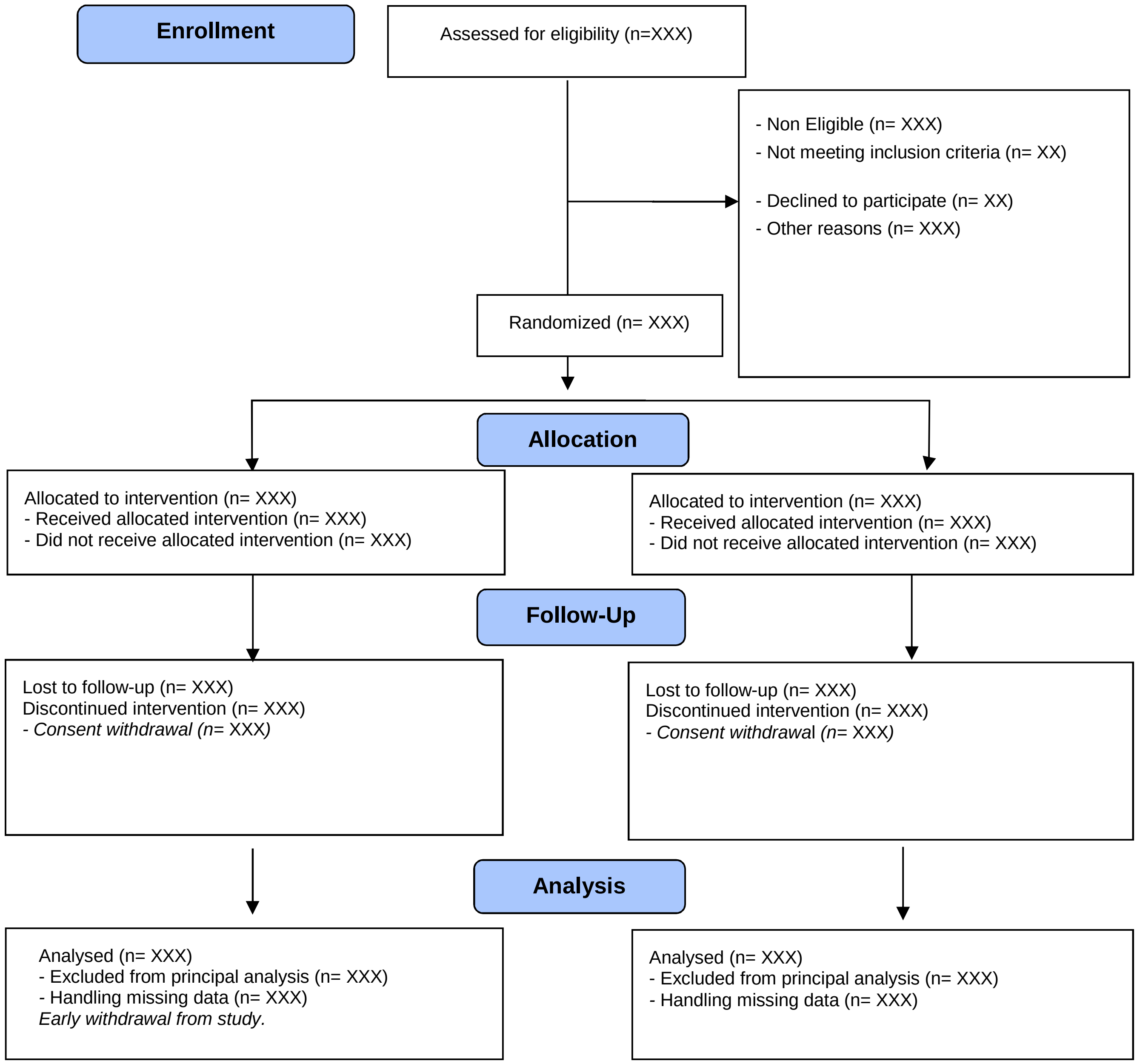

Supplement: S2 Text — (PDF) [file pmed.1002849.s003.pdf]
